# Identification and characterization of calreticulin as a novel plasminogen receptor

**DOI:** 10.1016/j.jbc.2023.105465

**Published:** 2023-11-17

**Authors:** Alamelu G. Bharadwaj, Gillian C. Okura, John W. Woods, Erica A. Allen, Victoria A. Miller, Emma Kempster, Mark A. Hancock, Shashi Gujar, Rimantas Slibinskas, David M. Waisman

**Affiliations:** 1 Departments of Pathology, Dalhousie University, Halifax, Nova Scotia, Canada; 2 Biochemistry and Molecular Biology, Dalhousie University, Halifax, Nova Scotia, Canada; 3 McGill SPR-MS Facility, McGill University, Montréal, Québec, Canada; 4 Life Sciences Center, Institute of Biotechnology, Vilnius University, Vilnius, Lithuania

**Keywords:** plasminogen, plasmin, plasminogen regulation, calreticulin, tissue plasminogen activator

## Abstract

Calreticulin (CRT) was originally identified as a key calcium-binding protein of the endoplasmic reticulum. Subsequently, CRT was shown to possess multiple intracellular functions, including roles in calcium homeostasis and protein folding. Recently, several extracellular functions have been identified for CRT, including roles in cancer cell invasion and phagocytosis of apoptotic and cancer cells by macrophages. In the current report, we uncover a novel function for extracellular CRT and report that CRT functions as a plasminogen-binding receptor that regulates the conversion of plasminogen to plasmin. We show that human recombinant or bovine tissue–derived CRT dramatically stimulated the conversion of plasminogen to plasmin by tissue plasminogen activator or urokinase-type plasminogen activator. Surface plasmon resonance analysis revealed that CRT-bound plasminogen (*K*_D_ = 1.8 μM) with moderate affinity. Plasminogen binding and activation by CRT were inhibited by ε-aminocaproic acid, suggesting that an internal lysine residue of CRT interacts with plasminogen. We subsequently show that clinically relevant CRT variants (lacking four or eight lysines in carboxyl-terminal region) exhibited decreased plasminogen activation. Furthermore, CRT-deficient fibroblasts generated 90% less plasmin and CRT-depleted MDA MB 231 cells also demonstrated a significant reduction in plasmin generation. Moreover, treatment of fibroblasts with mitoxantrone dramatically stimulated plasmin generation by WT but not CRT-deficient fibroblasts. Our results suggest that CRT is an important cellular plasminogen regulatory protein. Given that CRT can empower cells with plasmin proteolytic activity, this discovery may provide new mechanistic insight into the established role of CRT in cancer.

Since its pivotal discovery as an intracellular calcium-binding protein (1) that was localized to the endoplasmic reticulum (ER) (2), calreticulin (CRT) has been shown to possess multiple functions in both physiological and pathological cellular processes. The best-established functions for intracellular CRT include roles as a protein chaperone (3, 4, 5, 6, 7) and regulating Ca^2+^ homeostasis (8, 9, 10, 11). Extracellular CRT has also been reported to play a role in other important functions, such as cell adhesion (12, 13, 14), migration (15), proliferation (15, 16, 17), and wound healing (16, 17, 18, 19). During cellular stress, CRT relocates to the extracellular surface, where it plays an important role in both innate and adaptive immunity. A role for extracellular CRT in phagocytosis was supported by the demonstration that CRT serves as a cell surface “eat-me” signal of dying cells and cancer cells (20, 21, 22, 23, 24). Thus, CRT exemplifies a danger-associated molecular pattern molecule that is released in association with tissue damage or injury and markedly increases the immunogenicity of dying cancer cells.

Considerable evidence in recent years has suggested that CRT dysfunction plays a role in cancer progression (25, 26, 27, 28). The stimulation of the translocation of intracellular CRT to the surface of cancer cells by irradiation or certain chemotherapeutic agent signals uptake by dendritic cells, a process referred to as immunogenic cell death (ICD). Cell surface CRT is exposed on cells that succumb to ICD but is lacking on the surface of cells that undergo non-ICD (24). The inhibition of CRT exposure by blocking antibodies or by CRT depletion abolishes the phagocytosis of tumor cells by dendritic cells and abrogates the immunogenicity of anthracycline-induced cell death (23, 24, 29). Of particular interest are reports of the involvement of CRT in the invasion of cancer cells (17, 27, 28, 30). For example, CRT overexpression increased the cell migration and invasion of BxPC-3 cells, while depletion of CRT resulted in the decreased migration and invasion of PANC-1 and Capan-2 cells (30). Both the phagocytosis of cancer cells by macrophages and cancer cell invasion are known to be regulated by the plasminogen/plasmin protease system (reviewed in (31, 32, 33)). Furthermore, the depletion of CRT from oral cancer cells resulted in the decreased activity of matrix metalloproteinase-2 (MMP-2) and MMP-9, possibly due to the downregulation of the FAK-ERK-MMP-2/MMP-9 signaling pathway (16). This study confirmed an earlier study that reported the loss of MMP-9 activity in CRT-depleted cells (34). However, MMP-2 and MMP-9 are secreted in an inactive form, and the conversion of this inactive form to an active form is also known to be regulated by the plasminogen/plasmin protease system (35, 36).

Plasminogen is a blood protein and zymogen of the protease plasmin. Plasminogen activators such as the tissue plasminogen activator (tPA) and the urokinase-type plasminogen activator (uPA) are released from cells and convert plasminogen into plasmin. This activation results from the cleavage of an Arg^561^-Val^562^ peptide bond within plasminogen which then gives rise to the active protease, plasmin. Plasmin cleaves fibrin, the major component of blood clots as well as certain extracellular matrix proteins. The activity of the plasminogen activators toward free plasminogen is very low; however, when plasminogen is bound to cell surface plasminogen receptors, this rate is dramatically stimulated (reviewed in (37, 38, 39, 40)).

Since multiple studies have shown the importance of both CRT and plasmin in several physiologically important processes, we have investigated the possibility that CRT might play a regulatory role in the plasminogen/plasmin protease system. For the first time, we report that CRT functions as a plasminogen-binding protein receptor that regulates plasmin generation at the cell surface. This pivotal observation presents the possibility that CRT-generated plasmin may play a role in several important plasmin-dependent processes, such as phagocytosis, and in multiple steps of cancer invasion and metastasis.

## Results

### Identification of CRT as a plasminogen regulatory protein

**Figure 1:**
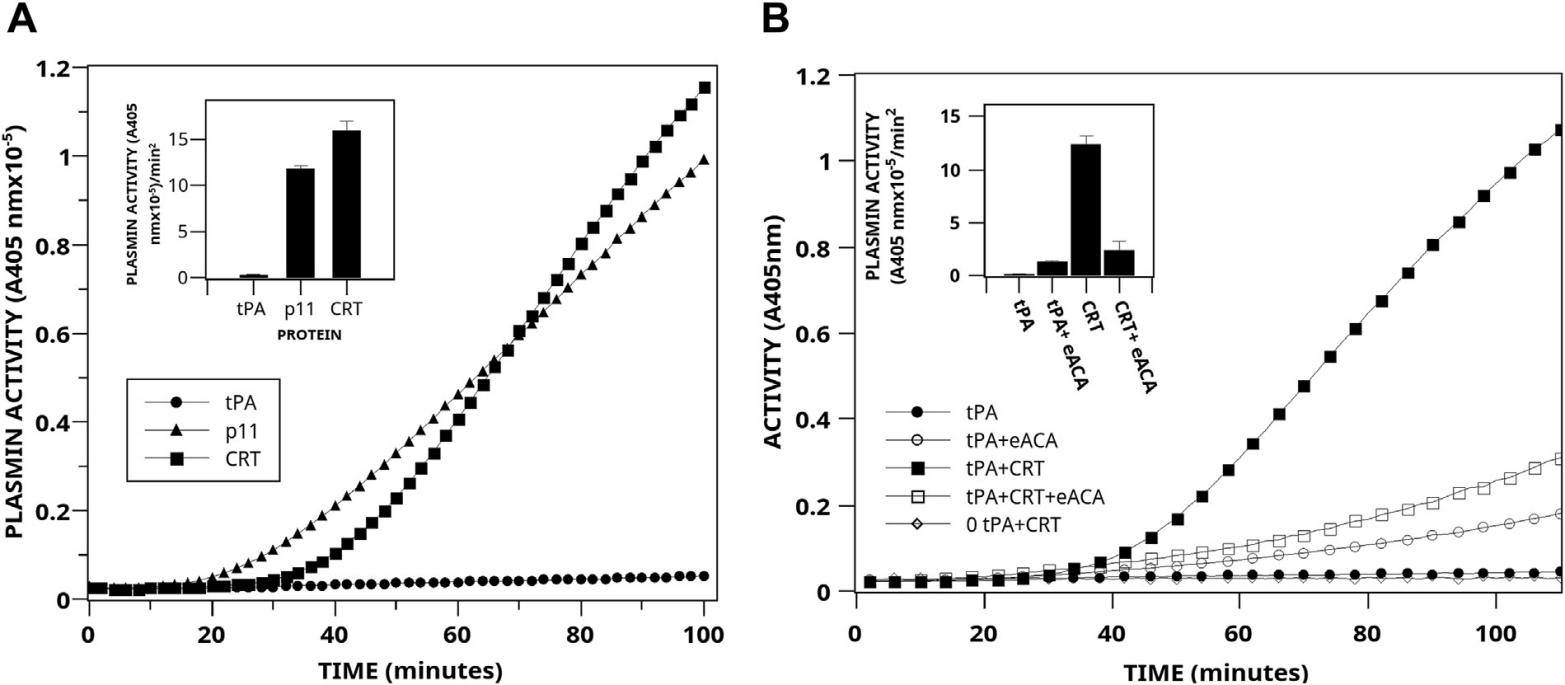
**Stimulation of plasmin generation by human recombinant CRT.***A*, time course of stimulation of tPA-dependent plasmin generation by recombinant human CRT. tPA (0.1 nM) was incubated at 37 °C in buffer A (50 mM Tris–HCl (pH 7.4), 50 mM NaCl, and 5 mM CaCl2) with 360 μM plasmin substrate in the absence (*circles*) or presence (*squares*) of 0.5 μM recombinant human CRT or 0.5 μM purified bovine lung S100A10 (p11) (*triangles*). The reaction was initiated by the addition of 0.16 μM Glu-plasminogen, and the reaction was monitored at 405 nm. The inset compares the rate of tPA-dependent plasminogen activation calculated from plots of *A*_405_ nm *versus* t^2^ (mean ± SD) in the absence or presence of 0.5 μM CRT or 0.5 μM S100A10 (p11) (see Fig. S1). *B*, inhibition of CRT activity by ε-ACA. tPA was incubated in the presence (*filled squares*) or absence (*filled circles*) of 0.5 μM human recombinant CRT as described above. In some experiments, 10 mM ε-ACA was added before the reaction was initiated (*open symbols*). The inset compares the rate of tPA-dependent plasminogen activation calculated from plots of *A*_405_ nm *versus* t^2^ (mean ± SD; n = 3). ε-ACA, epsilon-aminocaproic acid; CRT, calreticulin; tPA, tissue plasminogen activator.

To investigate the potential role of CRT in the plasminogen/plasmin system, we incubated human recombinant CRT with Glu-plasminogen and the plasminogen activator, tPA, and measured the rate of plasmin generation with an amidolytic plasmin substrate. Figure 1*A* compares the rates of activation of Glu-plasminogen by human recombinant CRT and the well-established plasminogen receptor, S100A10 (p11) (41, 42). We observed that the rate of tPA-dependent plasminogen activation increased from 0.30 ± 0.02 U (mean ± SD, n = 3) to 15.9 ± 1 U (mean ± SD, n = 3) in the presence of 0.5 μM CRT, an increase of 53-fold. Similarly, 0.5 μM recombinant human p11 displayed an activity of 11.75 ± 0.38 U (mean ± SD, n = 3) and an increase of 39-fold. As shown in Figure 1*B*, epsilon-aminocaproic acid (ε-ACA) inhibited plasminogen activation by CRT by about 80%. This suggests that CRT possesses a novel protein-binding domain for plasminogen, consisting of an internal lysine(s) residue that interacts with the lysine-binding domains (LBS) of plasminogen. Overall, these results suggested that CRT was a potent regulator of plasmin generation *in vitro*. The raw data showing the representative linearization of the reaction rates are shown in the Fig. S1.

**Figure 2:**
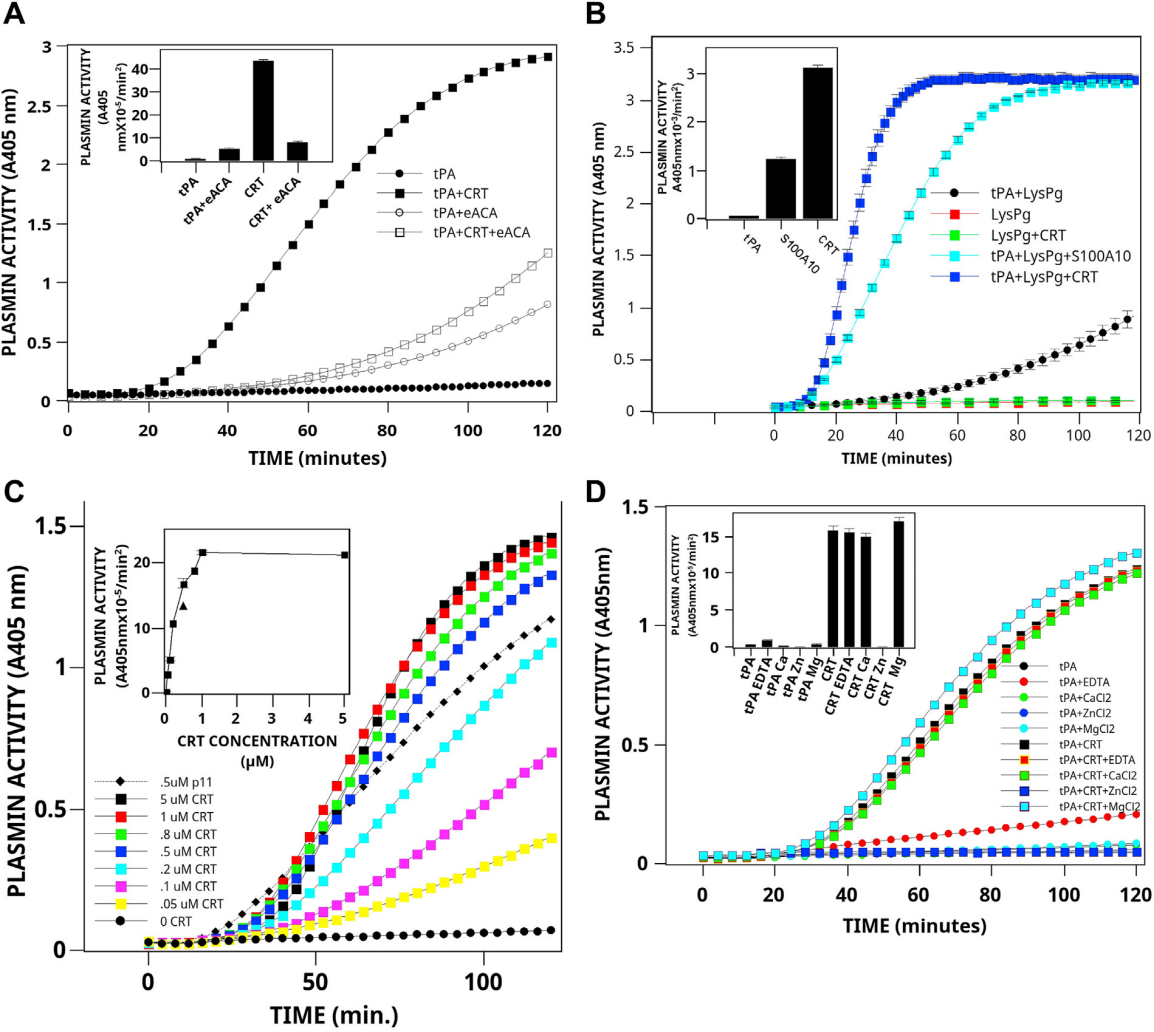
**Stimulation of plasmin generation by bovine liver CRT.***A*, time course of stimulation of tPA-dependent plasmin generation by purified bovine liver CRT. tPA (0.1 nM) was incubated at 37 °C in buffer A with 360 μM plasmin substrate in the absence (*filled circles*) or presence (*filled squares*) of 1 μM bovine liver CRT. Some reactions contained 1 mM ε-ACA (*open symbols*). The reaction was initiated by the addition of 0.16 μM Glu-plasminogen, and the reaction was monitored at 405 nm. The inset compares the rate of tPA-dependent plasminogen activation calculated from plots of A_405_ nm *versus* t^2^ (mean ± SD). *B*, activation of Lys-plasminogen by CRT. The reaction was conducted as detailed in *A*, except that the addition of 0.145 μM Lys-plasminogen initiated the reaction. *C*, concentration dependence. tPA (0.1 nM) was incubated at 37 °C in buffer A with 0.16 μM Glu-plasminogen, and 360 μM plasmin substrate in the presence of various concentrations of bovine liver CRT. The inset compares the rates of tPA-dependent plasminogen activation calculated from plots of *A*_405_ nm *versus* t^2^ (mean ± SD). The *triangle* depicts the rate for 0.5 μM S100A10. *D*, metal ion dependence. tPA (0.1 nM) was incubated at 37 °C in 50 mM Tris–HCl (pH 7.4), 50 mM NaCl with 360 μM plasmin substrate in the absence (*circles*) or presence (*squares*) of 1.0 μM bovine liver CRT and no addition, 5 mM EDTA, 5 mM CaCl_2_, 0.5 mM ZnCl_2_ or 5 mM MgCl_2_. The reaction was initiated by the addition of 0.16 μM Glu-plasminogen and monitored at 405 nm. The inset compares the rates of tPA-dependent plasminogen activation calculated from plots of *A*_405_ nm *versus* t^2^ (mean ± SD). ε-ACA, epsilon-aminocaproic acid; CRT, calreticulin; tPA, tissue plasminogen activator; uPA, urokinase-type plasminogen activator.

The recombinant CRT used in our experiments was expressed in *Escherichia coli* and corresponded to the amino acids 18 to 417 of human CRT and also contained an N-terminal His-tag. However, we could not rule out the possibility that this recombinant CRT, which was not full length, was not in the same conformation as cellular CRT. It was also possible that the purification of the recombinant CRT by Ni^2+^ chromatography resulted in the generation of a denatured protein. Denatured proteins can, in general, stimulate tPA-dependent plasminogen activation (43). To rule out this possibility, we purified native CRT from bovine liver to cross-validate our recombinant human CRT findings. As shown in Figure 2*A*, the purified bovine liver CRT dramatically stimulated tPA-dependent plasmin generation. The rate of tPA-dependent plasminogen activation increased from 0.21 ± 0.03 U (mean ± SD, n = 3) in the presence of tPA alone to 16.5 ± 0.48 U (mean ± SD, n = 3) in the presence of both tPA and 1 μM CRT, an increase of about 80-fold. Furthermore, as observed for the recombinant CRT, the addition of ε-ACA blocked CRT activity, suggesting that lysine residue(s) of CRT interacted with the LBS of plasminogen.

We also observed that the partially activated form of plasminogen, namely Lys-plasminogen, was also activated by CRT. Interestingly, at equimolar concentrations, CRT was a slightly more potent activator of Lys-plasminogen than the plasminogen receptor, S100A10 (Fig. 2*B*). The stimulation of tPA-dependent plasminogen activation by CRT was concentration-dependent (Fig. 2*C*). As little as 0.05 μM CRT produced a 10-fold stimulation of tPA-dependent plasmin generation. At saturating concentrations (1 μM), CRT stimulated plasminogen activation by about 80-fold. Interestingly, although CRT has been shown to interact with several metals, including Ca^2+^, Zn^2+^ (44), and Mg^2+^ (45), we observed that the ability of CRT to stimulate tPA-dependent plasminogen activation was not affected by Ca^2+^ or Mg^2+^ (Fig. 2*D*). The loss of CRT activity in the presence of Zn^2+^ was most likely due to the inhibition of tPA by this metal (46).

### CRT stimulates plasminogen activator–dependent conversion of plasminogen to plasmin

**Figure 3:**
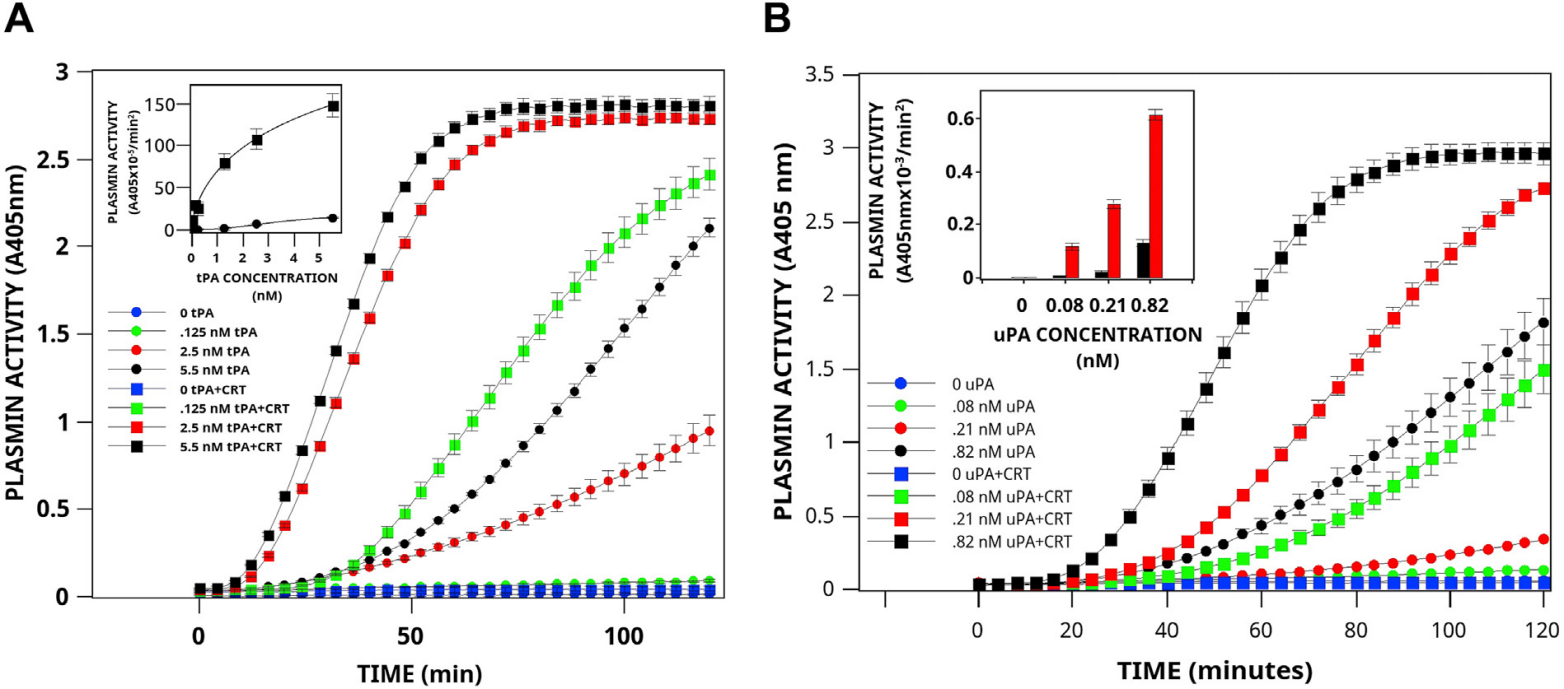
**CRT stimulates tPA- and uPA-dependent plasminogen activation.***A*, activation by tPA. CRT (1 μM) was incubated at 37 °C in buffer A with 360 μM plasmin substrate in the absence or presence of tPA (*squares*). Parallel reactions were conducted in the absence of CRT (*circles*). The reaction was initiated by the addition of 0.16 μM Glu-plasminogen, and the reaction was monitored at 405 nm. The inset compares the rates of tPA-dependent plasminogen activation calculated from plots of *A*_405_ nm *versus* t^2^ (mean ± SD). Titration data were analyzed by nonlinear least-*squares* curve fitting with the four-parameter logistic equation y = (a - d)/[1 + (x/c) b ] + d, where a is the asymptotic maximum, b is the slope parameter, c is the value at the inflection point (A_0.5_), and d is the asymptotic minimum. The convergent best fit for experiments conducted in the presence of CRT was determined for a = 353, b = 0.64, c = 9.26, and d = 2.16 (rms for the fit of 0.995). In contrast, the convergent best fit for experiments conducted in the absence of CRT was determined for a = 19.6, b = 1.48, c = 3.58, and d = 0.17 (root mean square for the fit of 0.999). *B*, activation by uPA. CRT (1 μM, *squares*) was incubated at 37 °C in buffer A with 360 μM plasmin substrate in the absence, or presence of 0.08 nM, 0.21 nM, or 0.82 nM single chain uPA (*squares*). Parallel reactions were conducted in the absence of CRT (*circles*). The reaction was initiated by the addition of 0.16 μM Glu-plasminogen and monitored at 405 nm. The inset compares the rates of uPA-dependent plasminogen activation calculated from plots of *A*_405_ nm *versus* t^2^ (mean ± SD). CRT, calreticulin; tPA, tissue plasminogen activator; uPA, urokinase-type plasminogen activator.

Streptokinase binds to plasminogen and induces a conformational change in plasminogen to an enzymatically active plasminogen–streptokinase complex (47). Therefore, streptokinase can activate plasminogen in the absence of plasminogen activators. We observed that CRT activity was dependent on the presence of tPA since, in the absence of tPA, CRT did not activate plasminogen (Fig. 3*A*). Furthermore, CRT activity was also dependent on the concentration of tPA. Kinetic analysis suggested that CRT affected the maximum velocity of tPA about 18-fold in the presence of plasminogen. However, it was unclear if CRT directly stimulated tPA activity or if the interaction of CRT with plasminogen increased the susceptibility of plasminogen to cleavage by tPA. However, we observed that the tPA amidolytic activity was minimally stimulated by CRT (Fig. S2).

The uPA has a relevant role in many physiological conditions, such as intravascular fibrinolysis, angiogenesis, tissue regeneration, and tumor progression, and has been shown to play a crucial role as a soluble or membrane-associated protease (48). We observed that when uPA was incubated with plasminogen and CRT, a robust stimulation of uPA-dependent plasminogen activation was observed (Fig. 3), and this effect was dose-dependent (Fig. 3*B* inset). As was observed for tPA, the uPA amidolytic activity was not stimulated by CRT (Fig. S3). One would expect that if CRT functioned to directly increase plasminogen activator activity, then the incubation of plasminogen activators, tPA, and uPA with CRT would increase their activity toward any substrate, including the amidolytic substrate. Since CRT did not stimulate their amidolytic activity, it is reasonable to suspect that CRT might affect their substrate, namely plasminogen. Our data, therefore, present the possibility that the interaction of CRT and plasminogen resulted in the conversion of plasminogen into a more easily activatable substrate for the plasminogen activators, tPA and uPA.

**Figure 4:**
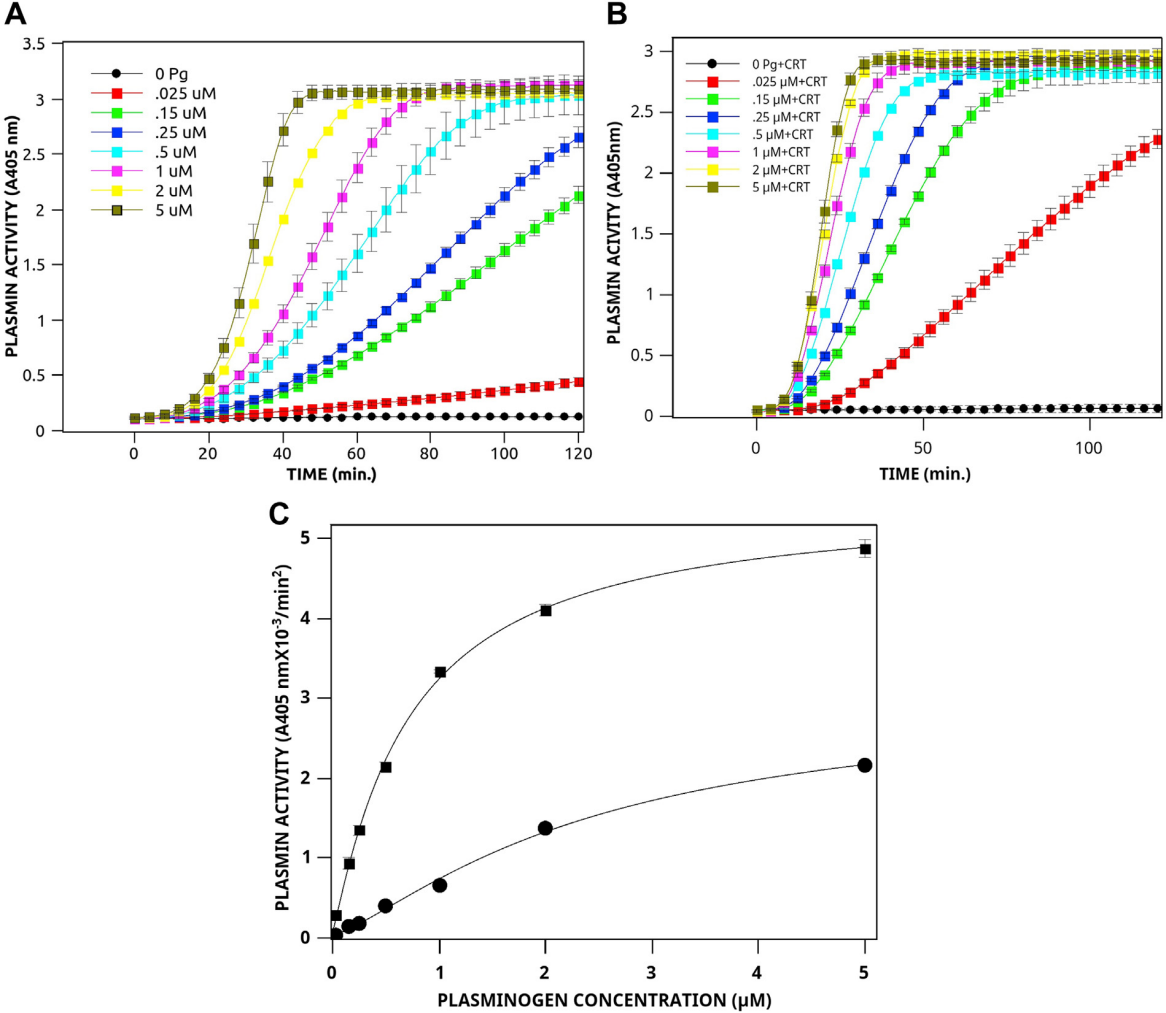
**Mechanism of stimulation of tPA-dependent plasminogen activation by CRT.** tPA (2.5 nM) was incubated at 37 °C in buffer A with 360 μM plasmin substrate in the absence (*A*) or presence (*B*) of 1.0 μM bovine liver CRT in the presence of several concentrations of Glu-plasminogen. *C*, the value for k was calculated according to Ref. (106) using the equation: *A*_405_ nm = 0.5Δε405 k_1_ k[t-PA]t^2^, where Δε405 = 10,500, [tPA] = 5.6 nM, and k_1_, the plasmin turnover number was calculated from a standard curve of plasmin amidolytic activity as 7.29 s^−1^. Titration data were analyzed by nonlinear least-squares curve fitting with the equation y = kcat/[1 + (x/A_0.5_) ^n^ ], as described in Figure 3. The values for the curve fit were kcat = 3.88 × 10^−3^, A_0.5_ = 2.43, n = 1.31 for tPA, and kcat = 7.12 × 10^−3^, A_0.5_ = 0.73 and n = 1.08 for tPA and CRT. CRT, calreticulin; tPA, tissue plasminogen activator.

The kinetic parameters for plasminogen activation by tPA are presented in Figure 4. Since tPA has only slight activity against plasminogen in the absence of plasminogen receptors, the determination of the kinetic constants for plasminogen activation in the absence of CRT required higher concentrations of tPA. Therefore, data is presented for a tPA concentration of 5.6 nM, which is about 40-times the physiological concentration (0.14 nM) (49) (Fig. 4*C*). We observed that CRT stimulated the tPA-dependent activation of Glu-plasminogen by producing a small increase in the kcat and a large decrease in the A_0.5_. This resulted in an increase in the catalytic efficiency of plasminogen activation of about 6-fold under these experimental conditions. These data are consistent with CRT inducing a conformational change in plasminogen, resulting in a conformation that is more easily activatable by tPA.

### Identification of CRT as a plasminogen-binding protein

**Figure 5:**
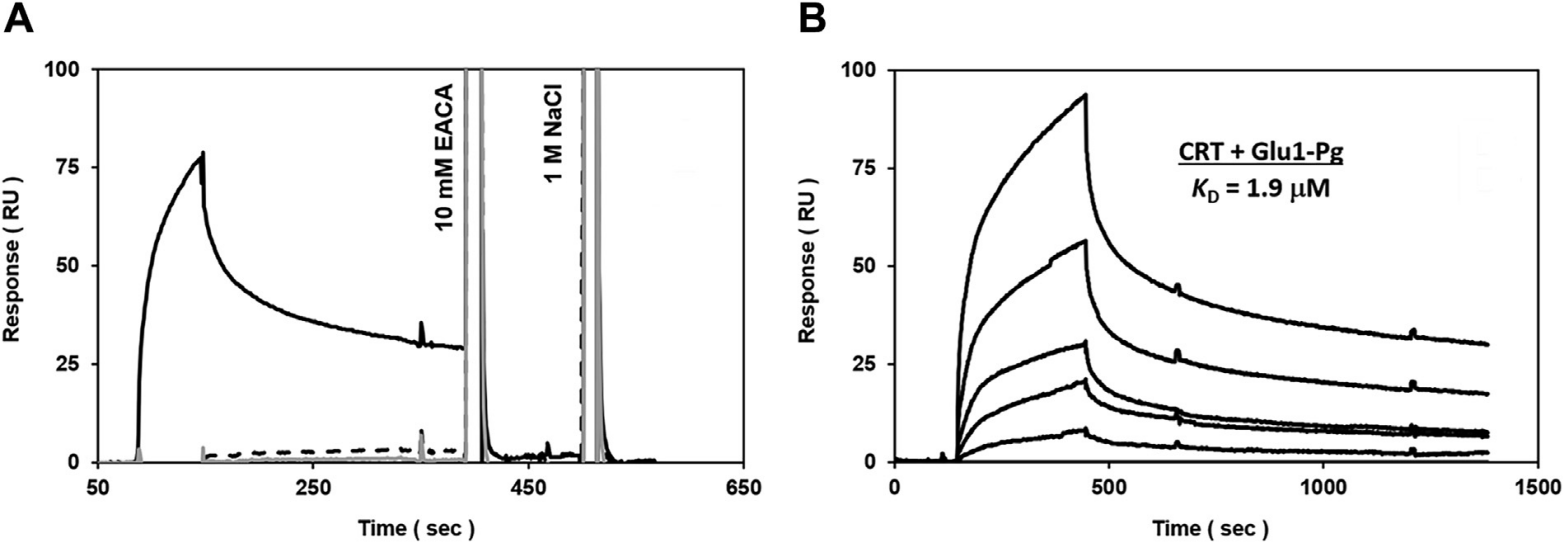
**Specific, dose-dependent binding between CRT and Glu-plasminogen is lysine-dependent, as assessed by SPR.***A*, representative specificity of buffer blank (*gray line*, *baseline*), 500 nM bovine serum albumin (*dashed black line*, negative control), or 500 nM Glu-plasminogen (*solid black line*) injected over immobilized CRT surfaces at 50 μl/min (1 min association + 3 min dissociation). To remove residually bound plasminogen (*black arrows*), the immobilized surfaces were readily regenerated using a lysine analog (spike #1, 10 mM epsilon-amino caproic acid) prior to an added high-salt pulse (spike #2, 1 M NaCl). *B*, representative kinetics for increasing plasminogen concentrations (0, 0.031, 0.062, 0.125, 0.25, 0.5, and 1 μM) titrated over immobilized CRT surfaces at 50 μl/min (5 min association + 15 min dissociation). Figure 5*B* inset, dose-dependent isotherm for plasminogen binding to CRT (*black symbols*) was subjected to nonlinear regression analysis (*gray line*) to predict the equilibrium dissociation constant (K_D_ = 1.9 ± 0.4 μM). CRT, calreticulin; SPR, surface plasmon resonance.

To test for direct binding between plasminogen and CRT, we performed label-free, real-time surface plasmon resonance (SPR). The purified ligands, bovine CRT (test receptor), or human recombinant S100A10 (positive control), were amine-coupled to SPR sensors so the purified human plasminogen “analyte” could be titrated over both receptors simultaneously. The binding of Glu-plasminogen to immobilized CRT was specific (compared to a negative response with bovine serum albumin) and, notably, lysine-dependent, as demonstrated by the simplistic regeneration using a low 10 mM concentration of the lysine analog ε-ACA (Fig. 5*A*). When titrated in a dose-dependent manner, the equilibrium dissociation constant for plasminogen binding to CRT was determined to be appK_D_ of 1.9 μM, which was slightly weaker than S100A10 (appK_D_ of 0.5 μM) (Fig. S4).

### The carboxyl-terminal domain contributes to plasminogen activation

**Figure 6:**
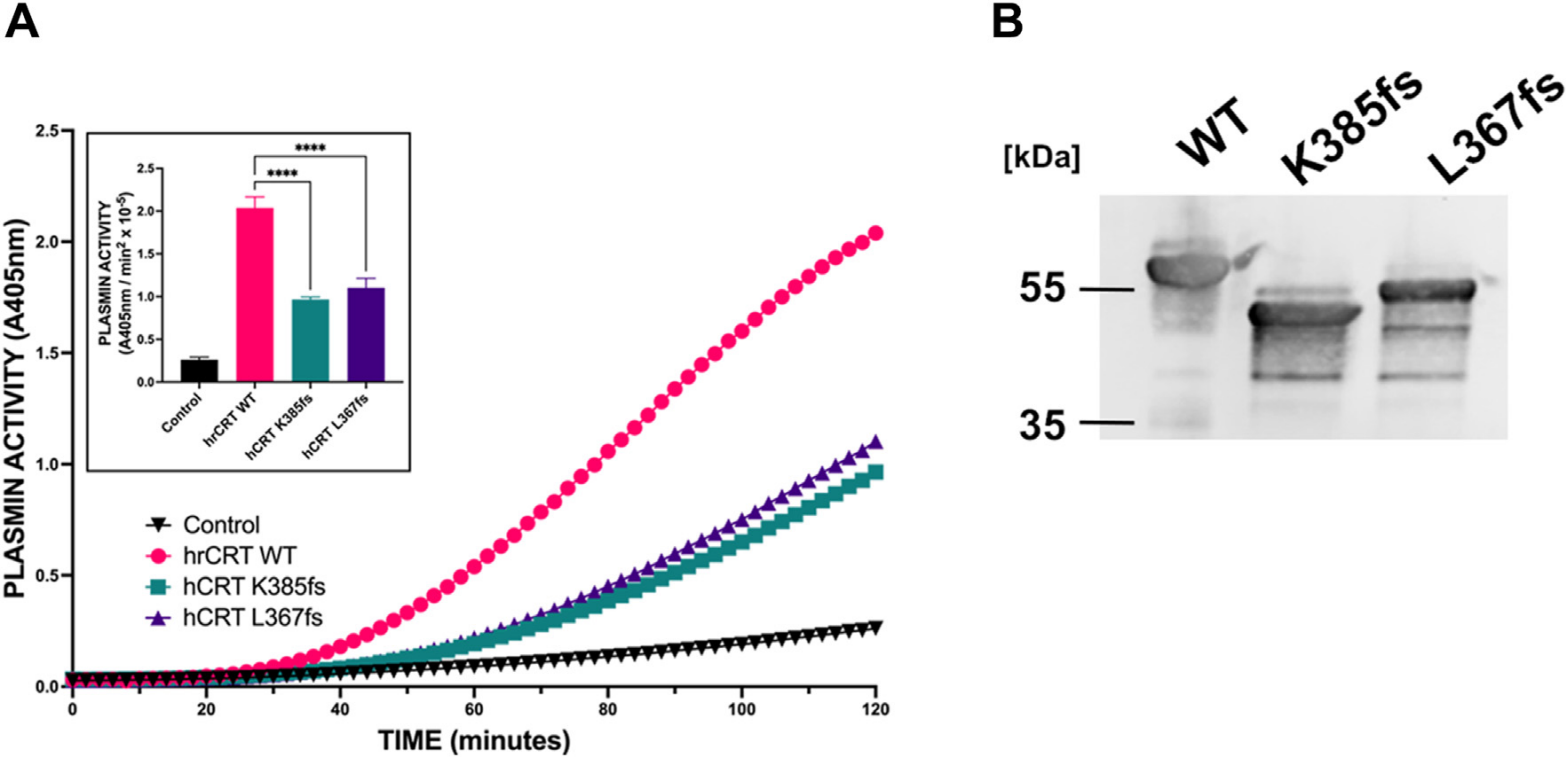
**Analysis of plasminogen activation by calreticulin mutants.** We examined two frameshift mutations of CRTfs-L367, and CRTfs-K835, which have been identified in some patients with MPN. We obtained human recombinant CRT WT and two mutants, CRTfsL367 (Del52) and CRTfs-K835 (Ins5) expressed and purified from *Saccharomyces cerevisiae* by the Slibinskas laboratory. *A*, plasmin generation: WT (*pink spheres*) and mutant CRT (*blue squares* and *purple triangles*) (1 μM) were incubated at 37 °C in buffer A with 360 μM plasmin substrate in the presence of tPA (*squares*). Parallel reactions were conducted in the absence of CRT (*black spheres*). The reaction was initiated by the addition of 0.16 μM Glu-plasminogen, and the reaction was monitored at 405 nm. The inset compares the rates of tPA-dependent plasminogen activation calculated from plots of *A*_405_ nm *versus* t^2^ (mean ± SD). *B*, the purity and size of the yeast CRT WT and mutants was examined by SDS-PAGE and immunoblotting with CRT antibody (Ab92516). Significance was determined by one-way ANOVA with multiple comparisons. Significant *p* values are indicated as follows: ∗ = < 0.05, ∗∗ = < 0.01, and ∗∗∗ = < 0.001. CRT, calreticulin; MEF, mouse embryonic fibroblast; MPN, myeloproliferative neoplasm; tPA, tissue plasminogen activator.

Somatic frameshift mutations in exon 9 of CRT are the second most common alteration after the JAK2V617F mutation in the myeloproliferative neoplasms, essential thrombocythaemia, and primary myelofibrosis (50, 51). Two frameshift mutants that have been reported are del52 (also called CRTfsL367), which has a deletion of 52 nucleotides, and Ins5 (also called CRTfsK385), which has an insertion of five nucleotides. These mutations result in a +1 frameshift and a novel carboxyl terminus. The Slibinskas laboratory has expressed these human recombinant mutants in *Saccharomyces cerevisiae* and detailed their structure and stability (52). We examined two frameshift mutations of CRTfs-L367 and CRTfs-K835, which have been identified in some patients with myeloproliferative neoplasm. We observed that compared to the WT CRT, both mutants were much less active and demonstrated a 50 to 60% loss in plasminogen activation activity (Fig. 6). This suggested that the carboxyl-terminal domain of CRT played an important role in plasminogen activation.

### CRT-deficient cells produce less plasmin

**Figure 7:**
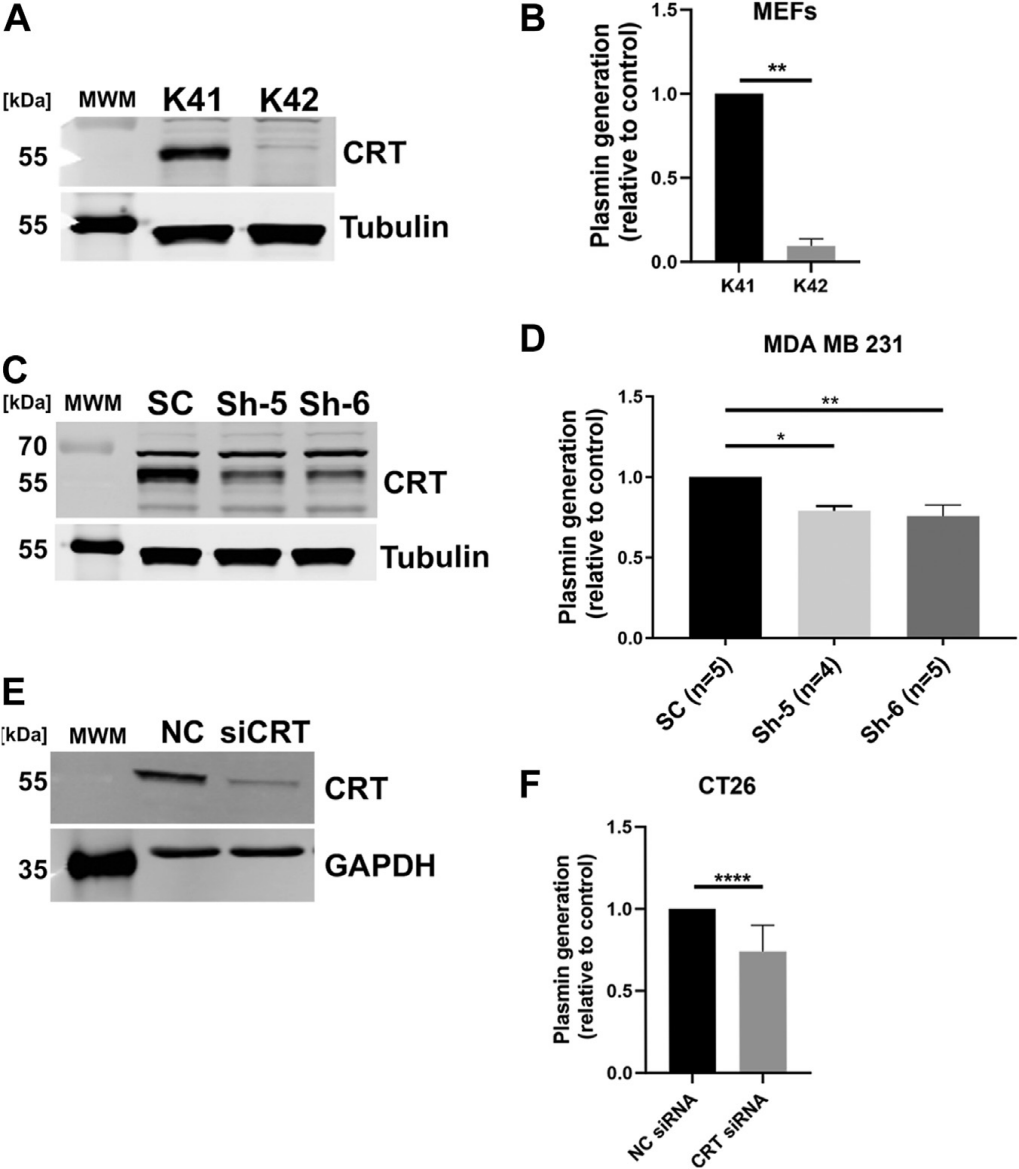
**Plasminogen activation by CRT-deficient cells.***A*, CRT-WT (K41) and CRT-KO MEFs were obtained from the Michalak lab and cell lysates were prepared as described in the Methods for immunoblotting. The expression of CRT was probed using rabbit anti-CRT antibody (Ab92516, Abcam). *B*, K41 and K42 were plated in a 96-well plate (20,000–30,000 cells per well) and incubated overnight. After incubation, the cells were washed and incubated with plasminogen (0.5 μM) for 30 min at room temperature, followed by the addition of substrate. The rate of appearance of the plasmin substrate cleavage product was measured spectrophotometrically at 405 nm. The rate of plasmin generation was determined from the slope of the *A*_405_ nm *versus* time^2^ progress curve (N = 4–6). Statistical significance was determined using an unpaired *t* test. *C*, CRT was depleted from MDA MB 231 using GIPZ lentiviral particles expressing CRT shRNA (sh5 and sh6) as described in the Methods. Cell lysates were prepared as described in the Methods. The expression of CRT was probed using rabbit anti-CRT antibody (Ab92516, Abcam). *D*, the selected population of CRT-depleted MDA MB 231 (sh6 and sh5) cells was seeded in a 96-well plate (40,000 cells/well) and incubated overnight. After incubation, the cells were washed and incubated with plasminogen (0.5 μM) for 30 min at room temperature, followed by the addition of substrate. The rate of appearance of the plasmin substrate cleavage product was measured spectrophotometrically at 405 nm. The rate of plasmin generation was determined from the slope of the *A*_405_ nm *versus* time^2^ progress curve (N = 4–6). Statistical significance was determined using one-way ANOVA (GraphPad Prism). *E*, CRT was depleted in CT26 mouse colon carcinoma cells using pooled siRNA sequences. The cell lysates were prepared and probed with anti-CRT antibody as described above. *F*, for measuring the plasminogen activation in the CRT-depleted CT26 cells, cells were transfected in a 96-well plate, as per the manufacturer’s instructions. The transfected cells were assayed for plasmin generation 72 h post transfection. Significance was determined by *t* test for experiments with the MEFs and CT26 cells and one-way ANOVA with multiple comparisons for MDA MB 231 cells. Significant *p* values are indicated as follows: ∗ = < 0.05, ∗∗ = < 0.01, and ∗∗∗ = < 0.001. CRT, calreticulin; MEF, mouse embryonic fibroblast.

Knock out of CRT by homologous recombination is embryonic lethal due to faulty cardiac organogenesis (53). Therefore, a CRT-KO mouse is not available to study CRT function. Alternatively, mouse embryonic fibroblasts (MEFs), obtained from WT (K41) or CRT-KO embryos (K42), have been widely used by many laboratories to study CRT function (54, 55, 56). When we incubated K41 and K42 MEF with plasminogen and measured plasmin generation, we observed a dramatic 90% loss in the capability of the CRT-KO MEF (K42) to generate plasmin (Fig. 7, *A* and *B*). We also generated CRT-depleted MDA MB 231 and CT26 cells using lentiviral shRNA particles and siRNA sequences, respectively. Measurement of plasmin generation in CRT-depleted MDA MB 231 cells (Sh5 and Sh6) resulted in a marginal (20–25%) but significant decrease in plasmin activity (Fig. 7, *C* and *D*). The loss of CRT in CT26 also resulted in a significant 25% decrease in plasmin activity (Fig. 7, *E* and *F*).

**Figure 8:**
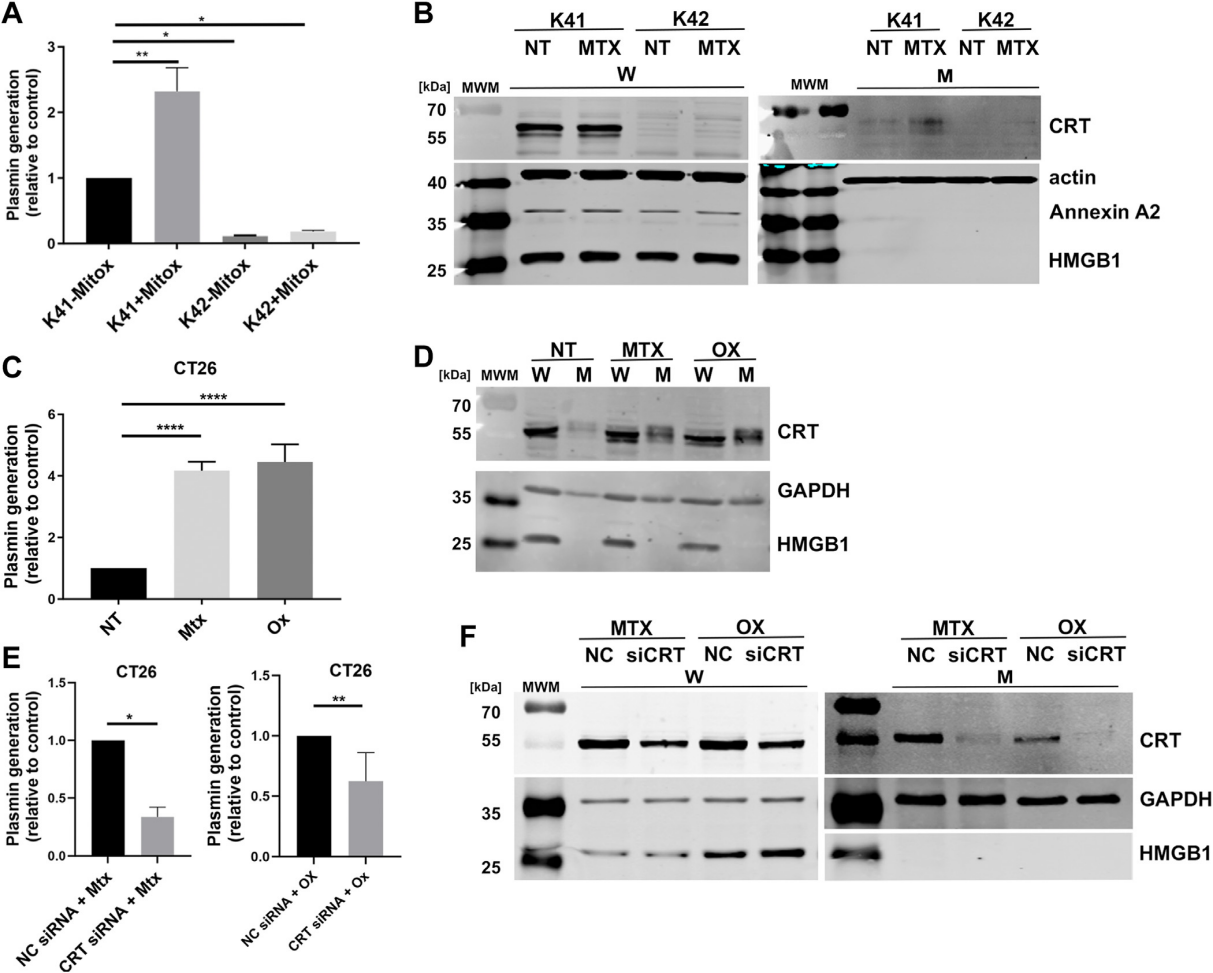
**Mitoxantrone treatment of K41 (WT) MEFs induces translocation of CRT to the cell surface and enhances plasmin generation.***A*, plasmin generation by MEFs. CRT-WT (K41) and CRT-KO MEFs (K42) (gift from Dr Michalak, U of Alberta) were plated in a 96-well plate (20,000–30,000 cells per well) and incubated overnight. The cells were treated with 5 μM mitoxantrone for 4 h. After incubation, the cells were washed and incubated with plasminogen (0.5 μM) for 30 min at room temperature, followed by the addition of substrate. The rate of appearance of the plasmin substrate cleavage product was measured spectrophotometrically at 405 nm. The rate of plasmin generation was determined from the slope of the *A*_405_ nm *versus* time^2^ progress curve (N = 4–6). *B*, the MEFs were treated with mitoxantrone for 4 h, and cells were biotinylated as described in the Methods. The biotinylated proteins were pulled down using Streptavidin-Sepharose beads, eluted using two sample buffer, and separated by SDS PAGE. CRT and control proteins (actin, HMGB1, and annexin A2) were probed in whole-cell lysate (W) and membrane (M) fractions. *C*, CT26 cells were plated in a 96-well plate and incubated overnight. The cells were then treated with 1 μM of mitoxantrone and 300 μM of oxaliplatin for 20 h, which was followed by measurement of plasmin activity as described above. *D*, the CT26 cells were treated with mitoxantrone and oxaliplatin in 100 mm dishes as described in (*C*) above, and cells were biotinylated as described in the Methods. The biotinylated proteins were pulled down using Streptavidin-Sepharose beads, eluted using 2× sample buffer, and separated by SDS-PAGE. CRT control proteins (GAPDH and HMGB1) were probed in whole-cell lysate (W) and membrane (M) fractions. *E*, CRT was depleted in CT26 cells plated in 96-well dishes using siRNA sequences as per the manufacturer’s instructions. The CRT-depleted cells (CRT siRNA) and nontargeting control siRNA-treated cells (NC siRNA) were treated with mitoxantrone (1 μM) and oxaliplatin (300 μM) for 20 h (48 h posttransfection) and plasmin activity was assayed. *F*, CRT-depleted and control siRNA (NC) transfected cells were biotinylated in 100 mm dishes as described in the Methods, and biotinylated proteins were precipitated using Streptavidin-Sepharose beads as described and separated by SDS-PAGE. CRT and control protein (GAPDH and HMGB1) were probed in whole-cell lysate (W) and membrane (M) fractions. Statistical significance was determined by one-way ANOVA with multiple comparisons for the MEFs and CT26 cells. Significance was determined by *t* test for experiments with CRT-depleted CT26 cells. Significant *p* values are indicated as follows: ∗ = < 0.05, ∗∗ = < 0.01, and ∗∗∗ = < 0.001. CRT, calreticulin; MEF, mouse embryonic fibroblast.

Since CRT functions as a chaperone in the ER that promotes the proper folding of proteins, we could not distinguish between the direct effects of CRT on plasmin generation or indirect effects that could be attributable to a stabilization effect on other plasminogen receptors. To investigate these possibilities, we took advantage of the known ability of cytotoxic antibiotics such as mitoxantrone and oxaliplatin to increase the extracellular levels of CRT in the MEFs and CT26 cells (24). The increase in cell surface CRT by short-term exposure to mitoxantrone occurs before apoptosis is activated (57). As shown in Figure 8, short-term exposure to mitoxantrone causes a dramatic increase in plasmin generation by K41 MEF, with a marginal effect in the K42 MEF (Fig. 8*A*). Mitoxantrone treatment resulted in increased cell surface CRT translocation in the K41 MEF but not in the K42 MEF, suggesting that the increase in plasmin generation is directly contributed by the cell surface CRT pool (Figs. 8*B* and S5). Similarly, in CT26, we observed a 4-fold increase in plasmin activity upon treatment with both mitoxantrone and oxaliplatin (Fig. 8*B*) with a concomitant increase in cell surface CRT (Fig. 8*D*). To further determine if this increase in plasmin activity can be eliminated with depletion or knockdown of CRT in CT26 cells, we used siRNA sequences to knockdown CRT and treated the cells with mitoxantrone and oxaliplatin. Loss of CRT suppressed the increase in plasmin activity observed in control cells treated with mitoxantrone and oxaliplatin (Fig. 8*E*) due to depletion of cell surface CRT (Fig. 8*F*).

## Discussion

Michalak and co-workers originally proposed that CRT was discovered by MacLennan's laboratory (58) as a Ca^2+^-binding protein of the rabbit skeletal muscle sarcoplasmic reticulum that was called the high-affinity Ca^2+^-binding protein (59). Although multiple laboratories have documented and described, in detail, the Ca^2+^-binding proteins of skeletal muscle sarcoplasmic reticulum, surprisingly, none of these reports have identified the high-affinity calcium-binding protein/CRT in the skeletal muscle sarcoplasmic reticulum (60, 61, 62, 63, 64, 65, 66, 67) (reviewed in (68)). Furthermore, we have reported that the bioinformatic analysis of the amino acid composition for the high-affinity Ca^2+^-binding protein (69), published by MacLennan’s laboratory, does not match CRT but matches the ADP-ribose deacetylase, MACROD2 (68). These data firmly support the notion that CRT and the high-affinity calcium-binding protein are distinct proteins.

The first report describing CRT was that of the protein called calreticulin (1, 2). These studies identified calreticulin as a liver Ca^2+^-binding protein of molecular weight 63,000 by SDS-PAGE or 55,000 by sedimentation equilibrium. We also showed that calreticulin localized to the ER (1, 2) and published the amino-terminal sequence of the protein (70). Subsequent studies completed the sequencing of the protein (59, 71) and identified CRT as an ER lumenal protein that functioned as a vital chaperone protein and as a regulator of Ca^2+^ homeostasis (60, 72, 73). CRT was later shown to be transported to the extracellular surface, where it performs multiple functions, including acting as an eat-me signal for phagocytosis and as a regulator of cancer cell migration and invasion (22, 23, 25, 74).

CRT possesses a lectin domain that recognizes a monoglucosylated oligosaccharide consisting of Glc_1_Man_9_GlcNAc_2_ (75, 76, 77). CRT also binds to asialoglycoproteins (74) and possesses a glycan-independent binding domain that binds certain polypeptides *via* hydrophobic interactions (76). For the first time in the current study, we (i) identify a novel function for extracellular CRT as a plasminogen receptor, (ii) show that CRT can regulate the tPA- and uPA-dependent activation of plasminogen to plasmin, and (iii) identify an interaction between plasminogen and CRT that involves the interaction on CRT internal lysines with the LBS of plasminogen.

The zymogen, Glu-plasminogen, is converted to the active serine protease, plasmin, through tPA or uPA-mediated cleavage in its activation loop domain (between Arg^561^ and Val^562^). The best-described physiological function of plasmin is blood clot fibrinolysis and restoring normal blood flow (48, 78). Plasmin also plays roles in phagocytosis (29, 79, 80) and inflammation and wound healing (40, 81). Plasmin plays a critical role during the multiple steps of cancer invasion and metastasis by participating in the degradation of several extracellular matrix proteins and activating certain growth factors, resulting in aggressive cancers (33, 82, 83). The plasmin involved in these diverse processes is generated at the cell surface by a process that is highly regulated by a group of cell-surface proteins called plasminogen receptors. These plasminogen receptors not only function to stimulate tPA- and uPA-dependent plasminogen activation and to localize plasmin proteolytic activity to the cell surface but also to protect both the plasminogen activators and plasmin from rapid inactivation by the abundant inhibitors that surround cells (37, 40, 84). Plasminogen receptors are broadly distributed on both eukaryotic and prokaryotic cell types, and most cells have a high capacity for binding plasminogen. Typically, the affinity for plasminogen binding by cells ranges between 0.5 to 2 μM (38).

The lysine mimetic ε-ACA effectively blocks the binding to and enhancement of plasminogen activation on eukaryotic cell surfaces. Interestingly, treatment of U937 cells with carboxypeptidase B (CpB) reduced plasminogen binding by about 60% while reducing plasminogen activation by about 95% (85). Since CpB proteolytically removes lysine and arginine residues from the carboxyl terminal of proteins, it was concluded that only a subset of plasminogen receptors, those that have carboxyl-terminal lysine residues, participate in plasminogen activation. This observation was consistent with the reported binding of lysine to the cationic center of the lysine-binding kringle domains since only the carboxyl-terminal lysine of the plasminogen receptor possesses a free carboxylate group. Plasminogen receptors that possess a carboxyl-terminal lysine include α-enolase, cytokeratin 8, S100A10, TIP49a, histone H2B, and PlgR_kt_. However, a second group of CpB-insensitive plasminogen receptors that utilize internal lysine(s) for plasminogen binding and activation have been reported and include tissue factor, glucose-regulated protein-78, αVβ3, αMβ2, and αIIbβ3 (38, 39). CRT does not possess a carboxyl-terminal lysine, but its ability to bind plasminogen and stimulate plasminogen activation is inhibited by ε-ACA (Fig. 1). This suggests that an internal lysine(s) of CRT interacts with the plasminogen LBS. Multiple studies have suggested that plasminogen receptors that utilize internal lysine residues to facilitate plasminogen activation can play a major role in cellular plasminogen activation (86, 87, 88).

Since plasmin and CRT have overlapping functions as regulators of multiple physiological processes, we explored the possibility that CRT might function as a plasminogen receptor. To test the possibility that CRT might be a plasminogen regulatory protein, we compared the rates of plasmin generation in the absence or presence of CRT (*versus* S100A10 as a positive control) using *in vitro* plasminogen activation assays. We observed that the rates of tPA-dependent plasminogen activation were increased in the presence of CRT (or S100A10) and could be attenuated in the presence of the lysine analog ε-ACA, thus indicating that CRT may be a potent regulator of plasmin generation *in vitro*. Notably, recombinant human CRT or purified bovine liver CRT was a potent stimulator of tPA or uPA-dependent plasmin generation. Although CRT is a Ca^2+^-binding protein, we observed that metal ions were not required for the dramatic acceleration of tPA- or uPA-mediated plasminogen activation by CRT, establishing that plasmin regulation by CRT was Ca^2+^-independent.

Direct evidence for the function of CRT as a plasminogen receptor was the demonstration that CRT bound plasminogen with an apparent *K*_*D*_ of 1.9 μM (Fig. 5), well within the reported affinity for cellular plasminogen binding. For example, plasminogen binds to MDAMB231 cells with a K_d_ 1.8 μM (89). The presence of a plasminogen-binding site on CRT complements the other well-described binding domains on CRT. The predominant role of lectin-glycan interactions in the binding of CRT to cellular proteins has been well established, it is clear that lectin-independent associations between CRT and proteins do exist (76, 90). For example, SPR has been used to determine the affinity of CRT for several of these lectin-independent interactions, including rheumatoid arthritis shared epitope (11 μM) (91), complement C1q (0.5 μM) (92), glycosylated laminin (0.5 μM) (93), ERp29 (13 μM), and ERp57 (23 μM) (94). Therefore, the binding affinity of CRT for plasminogen is similar to other known ligands of CRT.

We also observed that although CRT dramatically stimulated the tPA- or uPA-mediated conversion of plasminogen to plasmin, it did not stimulate tPA or uPA activity toward a synthetic substrate. This suggests that CRT does not affect the enzymatic activity of these plasminogen activators and therefore presents the possibility that the interaction of CRT with plasminogen alters the conformation of plasminogen from its tight, poorly activatable conformation to a more open and more easily activatable conformation. Studies of other purified plasminogen receptors, such as S100A10, have also suggested that the interaction of plasminogen with plasminogen receptors results in conformational changes in plasminogen, which greatly facilitates its activation by plasminogen activators (95). When Glu-plasminogen binds to cells, its activation by tPA is markedly enhanced due to a reduction in the Km compared to plasminogen activation by tPA in solution (96). We observed that CRT also lowered the A_0.5_ of tPA for Glu-plasminogen and increased the k_cat_ *in vitro*. The net effect was an increased catalytic efficiency of tPA for its substrate plasminogen by approximately 6-fold. By comparison, fibrinogen fragments were shown to decrease the Km for tPA-dependent plasminogen activation from 5 μM to 0.1 μM (96).

The activation of uPA-dependent plasmin generation by CRT is particularly interesting because uPA, with its receptor, urokinase-plasminogen activator receptor, localizes to the lipid rafts and interacts with low density lipoprotein receptor-related protein 1, a major cell surface receptor for CRT (22, 97, 98). Furthermore, several plasminogen receptors, such as S100A10, histone H2B, and CRT, bind to phosphatidylserine at the cell surface. It is thought that these receptors are responsible for the increased plasminogen binding by apoptotic cells (39).

The plasminogen crystal structure has established that the kringle 1 lysine-binding site is the only LBS available for ligand binding in the compact form of circulating plasminogen, whereas all other LBS are blocked as they are engaged in intramolecular interactions, which results in the closed activation-resistant conformation of Glu-plasminogen (99). Certain plasminogen receptors bind to kringle 1 in Glu-plasminogen, resulting in a conformational change in the Glu-plasminogen and a conversion to a more open and more easily activatable molecule. Plasmin hydrolyzes the Lys^77^-Lys^78^ peptide bond of Glu-plasminogen, resulting in an alternative zymogen form called Lys-plasminogen. Lys-plasminogen is present in an open conformation, and its multiple kringle domains are available for binding. We have observed that CRT is also a potent stimulator of Lys-plasminogen, which suggests that CRT might promote an additional conformational change in the “open” conformation of Lys-plasminogen. This is an important observation for two reasons. First, it is thought that plasmin is generated by a sequence of events involving Glu-plasminogen binding to cells, its conversion to Lys-plasminogen, and its subsequent conversion to plasmin (100, 101). Therefore, the ability of CRT to convert Lys-plasminogen *in vitro* is evidence of a physiological role in plasmin generation *in vivo*. Second, this observation further supports the model that multiple CRT lysines might interact with multiple kringles of Lys-plasminogen. Furthermore, the observation that the two frameshift mutants, del52, and Ins5, demonstrate a significant loss in plasminogen activation activity suggests that the lysines that are lost in the mutated regions might be involved in plasminogen activation. Interestingly, the novel carboxyl terminus of these mutants is rich in positively charged amino acids, while the WT carboxyl terminus is rich in negatively charged amino acids. However, compared to the WT protein, the del52 and Ins5 mutants, the location of eight or four lysines, respectively, have been altered in the carboxyl terminus. The observation that the plasminogen activation activity loss of del52 and Ins5 are similar suggests the importance of the K385, K391, K401, and/or K414 in plasminogen activation.

To directly evaluate the role of CRT in cellular plasmin production, we utilized MEFs obtained from WT embryos (K41) and CRT-KO embryos (K42). Many laboratories have widely used these cell lines to study CRT function. Therefore, we incubated K41 and K42 embryonic fibroblasts with plasminogen and measured plasmin generation; we observed that the loss of CRT resulted in a dramatic 90% loss in plasmin generation. Similarly, we observed a marginal but significant decrease in plasmin generation in CRT-depleted breast cancer cells. To further validate these findings in another cell line, we used the CT26 colon carcinoma cells, which are routinely employed to examine the cell surface translocation and functionalities of CRT. These cells show a robust increase in cell surface CRT upon treatment with anthracyclines and are an ideal model to study the cell surface plasminogen receptor function of CRT. Loss of CRT in these cells showed a significant decrease in plasmin generation, whereas treatment with mitoxantrone and oxaliplatin resulted in a dramatic increase in plasmin activity and an increase in cell surface CRT. This result indicates that CRT is a regulator of plasmin generation *in cellulo*. However, the effects of CRT depletion followed by drug treatment, although significant, suggest the involvement of additional plasminogen receptors in the regulation of plasmin generation. This is not unexpected, as three plasminogen receptors are known to regulate plasmin-dependent macrophage movement (38).

To rule out the possibility that the loss of CRT might affect the levels of the well-established plasminogen receptors, we compared the levels of these receptors between the K41 and K42 fibroblasts. We were unable to observe any differences in the levels of these plasminogen receptors between these fibroblasts. However, it is unclear from our results if the loss of plasmin generation by CRT-deficient embryonic fibroblasts is due to the loss of CRT on the cell surface or due to the loss of the chaperone function of CRT, resulting in the decrease in the cell surface exposure of another, unknown, plasminogen receptor(s). However, certain cytotoxic antibiotics, such as anthracycline and mitoxantrone, are known to increase the extracellular levels of CRT (24, 57). The increase in cell surface CRT by short-term exposure to mitoxantrone occurs before apoptosis is activated. We observed that mitoxantrone exposure failed to activate plasmin generation in the K42 cells but produced a robust increase in plasmin generation in the K41 cells. This result supports the direct effect of CRT on plasmin generation.

## Conclusions

CRT is a novel plasminogen receptor that participates in the plasminogen activator-dependent conversion of plasminogen to plasmin. Plasmin is known to regulate many diverse processes, such as phagocytosis and cancer cell invasion, and is also known to be robustly generated by apoptotic cells. Future studies will be necessary to establish if the plasmin-generating activity of CRT plays a role in these processes.

## Experimental procedures

### Materials

Recombinant human CRT was obtained from Novus Biochemicals. Bovine liver CRT was purified according to (70) with modification. The procedure consisted of (NH_4_)_2_SO_4_ precipitation of bovine liver homogenates, followed by ion-exchange chromatography with diethylaminoethyl cellulose and Concanavalin A-Sepharose chromatography. Aliquots of the eluted fractions from Concanavalin A-Sepharose were assayed by SDS-PAGE and stained with Coomassie blue and Stains-All (2) and confirmed with an anti-CRT mAb (Abcam # ab2907, Sigma (HPA002242) and Abcam # ab92516, US Biologicals (C1036)). CRT used in these studies was >90% pure. Expression and purification of yeast WT recombinant human CRT and the frameshift mutants were as described (52). Human recombinant single-chain tPA was obtained from Genentech and further purified by chromatography on benzamidine-sepharose. Recombinant pro-uPA was obtained from Axxora. S100A10 was purified according to (95). Human Glu-plasminogen and Lys-plasminogen were obtained from Enzyme Research Laboratories. Unless specified otherwise, Glu-plasminogen was used in all experiments. Mitoxantrone (Cayman Chemical Company) and oxaliplatin (Tocris) were purchased from Cedarlane Labs.

### Cell culture

WT (K41 MEF) and CRT-deficient MEFs (K42 MEF) were a gift from Dr M. Michalak (University of Alberta) (53). MDA MB 231 cells were a kind gift from Dr Paola Marcato, Dalhousie University. The CT26 colon carcinoma cell line was a kind gift from Dr. Shashi Gujar. Cell lines were maintained at 37 °C and 5% CO_2_ in Dulbecco's modified Eagle's medium/F-12 medium (Thermo Fisher Scientific) supplemented with 10% fetal bovine serum (VWR) and 1% penicillin and streptomycin (Hyclone). All cells were tested for *mycoplasma* contamination using the Mycoalert kit (Lonza) and only mycoplasma-free cells were used in the experiments.

### Knockdown of CRT in cell lines

CRT was depleted in MDA MB 231 cells by lentiviral shRNA. Knockdown clones of CRT were generated using the pGIPZ vector (Dharmacon) packaged in HEK293T cells following standard protocols. Briefly, HEK293T cells were transfected using 4 μg GIPZ lentiviral CRT shRNA with Lipofectamine 2000. The virus-containing media was harvested 24 and 48 h after transfection for transduction in MDA MB 231 cells. Clones were selected by adding 2 μg/ml puromycin and subsequently maintaining 0.25 μg/ml puromycin media. For all knockdown clones created, a GIPz vector control clone (containing a scrambled nonspecific sequence in place of a shRNA) was generated simultaneously. CRT depletion was verified by Western blot analysis (anti-CRT, Abcam, Ab92516). Transient knockdown of CRT in CT26 cells was achieved by applying SMART Pool On Target siRNA sequences (Dharmacon) with Dharmafect 1 transfection reagent (Dharmacon) to cells as per the manufacturer’s protocol. The siRNA sequences are listed in Table S1.

### Cellular plasmin generation

Analysis of plasmin generation by cultured cells was performed as described (102). Cells (25,000 cells/well for MEFs), and 40,000 cells/well cells for MDA MB 231, and 30,000 cells/well for CT26)were seeded in 96-well plates overnight and washed three times with incubation buffer (Hank’s balanced salt solution containing 3 mM CaCl2 and 1 mM MgCl2). For drug treatments (mitoxantrone and oxaliplatin), the cells incubated overnight in the 96-well plate were treated with 5 μM of mitoxantrone for 4 h (MEFs) and 1 μM of mitoxantrone and 300 μM of oxaliplatin for 20 h before measurement of plasmin activity as follows. The adherent cells were then washed three times with Hank’s Balanced Salt Solution (Thermo Fisher Scientific) and incubated with 0.5 μM Glu-plasminogen (Molecular Innovations) for 30 min before the addition of 500 μM plasmin chromogenic substrate (H-D-val-leu-lys-pNA 2HCl) (Chromogenix, DiaPharma Group). Plasmin activity was measured spectrophotometrically (405 nm) every 2 min for 2 h (N = 6). Time course data are analyzed according to the equation describing the rate of p-nitroanilide production *A*_405_ nm = B + Kt^2^, where K is the rate constant for the acceleration of p-nitroanilide generation, t is time, and B is the *y*-intercept. Under our experimental conditions, K was proportional to the initial rate of plasmin formation from plasminogen. For transiently transfected CT26 cells, the cells were treated with the drugs 48 h posttransfection for 20 h, and plasmin activity was measured as described (103).

### *In vitro* plasminogen activation assay

The kinetics of tPA- or uPA-dependent plasminogen activation was determined by measuring the amidolytic activity of the plasmin generated during the activation of plasminogen as described previously (95). The reaction was performed at 37 °C in a 0.2 ml reaction mixture consisting of 50 mM Tris–HCl (pH 7.4), 50 mM NaCl, 5 mM CaCl2, 0.1 nM tPA, and 360 μM plasmin substrate (H-D-valyl-L-leucyl-L-lysine-p-nitroaniline dihydrochloride) (Molecular Innovations Novi). The addition of 0.16 μM Glu-plasminogen initiated the reaction, and the reaction progress was monitored at 405 nm in a Molecular Devices SpectraMAX M3 microplate reader. Initial rates of plasmin generation (*i.e.*, plasmin substrate cleavage) were calculated using linear regression analysis of plots of *A*_405_ nm *versus* time^2^ utilizing data points at a low extent of substrate conversion as outlined in (103). Titration data were analyzed by nonlinear least-squares curve fitting as described (103) with the four-parameter logistic equation y = (a-d)/[1 + (x/c) b] + d, where a is the asymptotic maximum; b is the slope parameter; c is the value at the inflection point (A_0.5_); and d is the asymptotic minimum. The nonlinear least-squares curve fitting was then iterated by allowing the three fitting parameters to float while utilizing the Marquardt method for the minimization of the sum of the squared residuals. The value for the tPA turnover number, k, was calculated from Glu-plasminogen titration curves according to the equation: A_405_ nm = 0.5Δε405 k_1_ k[tPA]t^2^, where Δε405 = 10,500, [tPA] = 5.6 nm, and k_1_, the plasmin turnover number was calculated from a standard curve of plasmin amidolytic activity as 7.29 s^−1^ (104). Data plots and analysis were performed with the open-source graphing program, QtiPlot (https://www.qtiplot.com).

### Amidolytic plasminogen activator assay

tPA amidolytic activity was directly measured at 37 °C in 0.2 ml of a reaction mixture consisting of 100 mM Tris–HCl (pH 8.4), 106 mM NaCl, and 0.1 g/L Triton X-100 and 8 nM of the tPA substrate, S-2288 (H-D-isoleucyl-L-prolyl-L-arginine-p-nitroaniline dihydrochloride-DiaPharma Group). The uPA amidolytic activity was measured in 0.2 ml of a reaction mixture consisting of 50 mM Tris–HCl (pH 7.4), 50 mM NaCl, 5 mM CaCl^2^, and 500 μM uPA substrate, DiaPharma 444-25). These reactions were monitored at 405 nm, and the reaction rate was calculated using linear regression analysis of plots of *A*_405_ nm *versus* time (in minutes). Typically, results are representative of at least three separate experiments performed in triplicate.

### Immunoblotting/Western blotting

Western blotting was employed to determine the total expression of CRT. Briefly, cells were lysed in radioimmunoprecipitation assay buffer with Halt protease and phosphatase inhibitor cocktail (Thermo Fisher Scientific) augmented with 1 mM PMSF. The prepared lysates were separated (25 μg) on 10% SDS-PAGE and transferred to nitrocellulose membranes. The membranes were blocked (Odyssey blocking buffer-Licor, Lincoln) and incubated with anti-CRT primary antibody (Abcam Ab2907 and Ab92516), anti-tubulin (Sigma, Canada), and anti-GAPDH (Santa Cruz Biotechnology Inc) primary antibodies overnight at 4 °C, and with IR-conjugated secondary antibody, DyLight 680/800 (Thermo Fisher Scientific) for 1 h. The stained membranes were scanned using the Odyssey scanning system (Licor).

### Biotinylation of cell surface proteins and immunoprecipitation

To evaluate cell surface translocation and expression of CRT, biotinylation of cell surface proteins was performed as described (105). Briefly, 10 to 20 × 106 cells in 100 mm dishes were washed with PBS containing 0.1 mM CaCl_2_ and 1 mM MgCl_2_ and biotinylated with 1.25 mg/ml Sulfo-NHS-Biotin (Thermo Fisher Scientific) for 30 min prepared in Biotinylation buffer (10 mM triethanolamine, 2 mM CaCl_2_, 150 mM NaCl, pH 7.5). The reaction proceeded for 30 min at 40 °C with gentle shaking before quenching by incubation for 20 min in PBS containing 0.1 mM CaCl_2_, 1 mM MgCl_2,_ and 100 mM glycine. The cells were then washed with PBS containing 0.1 mM CaCl2 and 1 mM MgCl_2_ and lysed in lysis buffer containing 1% Triton X-100, 150 mM NaCl, 5 mM EDTA, 50 mM Tris, pH 7.5 with protease, and phosphatase inhibitors. The lysates were homogenized, and 500 μg of protein was immunoprecipitated with Streptavidin-Sepharose beads (Thermo Fisher Scientific) overnight at 4 °C. The beads were washed with lysis buffer containing Halt protease and phosphatase inhibitor cocktail and 1 mM PMSF, and the biotinylated proteins bound to the beads were eluted by incubation for 10 min at 100 °C with 2× Laemmli sample buffer containing β mercaptoethanol. The proteins were separated on 10% SDS-PAGE and immunoblotted for CRT, actin (Sigma), annexin A2/p36 (BD Biosciences), GAPDH (Santa Cruz Biotechnology Inc), and HMGB1 (intracellular marker) (Sigma and AbCam). For drug-treated cells, surface biotinylation was performed as described 4 h after mitoxantrone treatment in MEFs and 20 h after mitoxantrone and oxaliplatin treatment in CT26 cells. In transiently transfected CT26 cells, surface biotinylation was performed 72 h posttransfection and 20 h post-drug treatment.

### Surface plasmon resonance

SPR experiments were performed on research-grade CM5 sensor chips at 25 °C using filtered (0.2 μm) and degassed HBS-ET buffer (10 mM Hepes, pH 7.4; 150 mM NaCl; 3 mM EDTA; 0.05% (v/v) Tween-20) with a BIACORE 3000 system (GE/Cytiva). Immobilized receptor surfaces were prepared using the Biacore Amine Coupling Kit as recommended by the manufacturer (10 μg/ml CRT in 10 mM sodium acetate pH 4 or 10 μg/ml S100A10 in 10 mM sodium acetate pH 5) at 1000 to 1300 resonance units (RU) final density. Corresponding reference surfaces were prepared in the absence of protein. Protein-grade detergents (Tween-20 and EMPIGEN) were from Anatrace; all other reagents were of analytical-grade quality.

To assess binding specificity, fixed 0.5 μM bovine serum albumin (negative control) and Glu-plasminogen were flowed (“KINJECT”) over reference and receptor-immobilized surfaces at 50 μl/min in multicycle mode (1 min association + 3 min dissociation). Between samples, the surfaces were regenerated at 50 μl/min using two 30 s pulses of solutions I (10 mM epsilon-amino caproic acid) and II (HBS-ET containing 1.0 M NaCl and 0.05% (v/v) EMPIGEN), followed by the “EXTRACLEAN” and “RINSE” procedures. To examine binding affinity, dose-dependent titrations of Glu-plasminogen (0–1 μM; 2-fold serial dilutions) were performed in a similar manner (50 μl/min x 5 min association + 15 min dissociation). SPR data were double-referenced and are representative of duplicate injections acquired from at least three independent trials. For each titration series, a buffer blank was injected first, the highest Glu-plasminogen analyte concentration second, and serial dilutions followed (from the lowest to the highest concentration repeated); comparing responses between the two highest analyte injections validated consistent immobilized surface activity throughout each assay. Experimental signal responses for Glu-plasminogen were also verified against the theoretical binding maxima predicted by the following equation: Rmax = MWA/MWL ∗ RL ∗ n where Rmax is the maximal binding response (RU) at saturating analyte concentration; MWA is the molecular mass (kDa) of the injected analyte; MWL is the molecular mass (kDa) of the immobilized receptor ligand; RL is the amount (RU) of the immobilized receptor, and n is the predicted binding stoichiometry (*e.g.*, 1:1). Since the multicycle titrations deviated from a simple “1:1 kinetic” binding model (most likely due to inherent sample heterogeneity, *e.g.*, Glu-Plasminogen isoforms), apparent equilibrium dissociation constants were determined by global fitting of the data (averaged responses at the end of each association phase plotted *versus* concentration, Req *versus* C) to the “steady-state affinity” model in BIAevaluation v4.1 (https://biaevaluation.software.informer.com).

### Statistical analysis

All statistical analyses were performed using Graph Pad Prism 10 (www.graphpad.com) and Qtiplot (https://www.qtiplot.com). When two experimental conditions were being compared, *t* tests were performed. For comparison between more than two conditions, one-way ANOVA and multiple comparison analysis were used. *p* values are represented as follows: ∗ = <0.05, ∗∗ = <0.01, ∗∗∗ = <0.001, and ∗∗∗∗ = <0.0001.

## Data availability

All data is contained within the manuscript.

## Supporting information

This article contains supporting information.

## Conflict of interest

The authors declare that they have no conflicts of interest with the contents of this article.

## References

[1] Waisman D.M., Smallwood J., Lafreniere D., Rasmussen H. (1984). Identification of a novel hepatic calcium-binding protein. Biochem. Biophys. Res. Commun..

[2] Waisman D.M., Salimath B.P., Anderson M.J. (1985). Isolation and characterization of CAB-63, a novel calcium-binding protein. J. Biol. Chem..

[3] Gelebart P., Opas M., Michalak M. (2005). Calreticulin, a Ca2+-binding chaperone of the endoplasmic reticulum. Int. J. Biochem. Cell Biol..

[4] Saito Y., Ihara Y., Leach M.R., Cohen-Doyle M.F., Williams D.B. (1999). Calreticulin functions in vitro as a molecular chaperone for both glycosylated and non-glycosylated proteins. EMBO J..

[5] Leach M.R., Cohen-Doyle M.F., Thomas D.Y., Williams D.B. (2002). Localization of the lectin, ERp57 binding, and polypeptide binding sites of calnexin and calreticulin. J. Biol. Chem..

[6] Nigam S.K., Goldberg A.L., Ho S., Rohde M.F., Bush K.T., MYu S. (1994). A set of endoplasmic reticulum proteins possessing properties of molecular chaperones includes Ca(2+)-binding proteins and members of the thioredoxin superfamily. J. Biol. Chem..

[7] Peterson J.R., Ora A., Van P.N., Helenius A. (1995). Transient, lectin-like association of calreticulin with folding intermediates of cellular and viral glycoproteins. Mol. Biol. Cell.

[8] Mery L., Mesaeli N., Michalak M., Opas M., Lew D.P., Krause K.H. (1996). Overexpression of calreticulin increases intracellular Ca2+ storage and decreases store-operated Ca2+ influx. J. Biol. Chem..

[9] Liu N., Fine R.E., Simons E., Johnson R.J. (1994). Decreasing calreticulin expression lowers the Ca2+ response to bradykinin and increases sensitivity to ionomycin in NG-108-15 cells. J. Biol. Chem..

[10] Liu H., Bowes R.C., van de Water B., Sillence C., Nagelkerke J.F., Stevens J.L. (1997). Endoplasmic reticulum chaperones GRP78 and calreticulin prevent oxidative stress, Ca2+ disturbances, and cell death in renal epithelial cells. J. Biol. Chem..

[11] Bastianutto C., Clementi E., Codazzi F., Podini P., De Giorgi F., Rizzuto R. (1995). Overexpression of calreticulin increases the Ca2+ capacity of rapidly exchanging Ca2+ stores and reveals aspects of their lumenal microenvironment and function. J. Cell Biol..

[12] Leung-Hagesteijn C.Y., Milankov K., Michalak M., Wilkins J., Dedhar S. (1994). Cell attachment to extracellular matrix substrates is inhibited upon downregulation of expression of calreticulin, an intracellular integrin alpha-subunit-binding protein. J. Cell Sci..

[13] White T.K., Zhu Q., Tanzer M.L. (1995). Cell surface calreticulin is a putative mannoside lectin which triggers mouse melanoma cell spreading. J. Biol. Chem..

[14] Fadel M.P., Dziak E., Lo C.M., Ferrier J., Mesaeli N., Michalak M. (1999). Calreticulin affects focal contact-dependent but not close contact-dependent cell-substratum adhesion. J. Biol. Chem..

[15] Hayashida Y., Urata Y., Muroi E., Kono T., Miyata Y., Nomata K. (2006). Calreticulin represses E-cadherin gene expression in Madin-Darby canine kidney cells via Slug. J. Biol. Chem..

[16] Chiang W.-F., Hwang T.-Z., Hour T.-C., Wang L.-H., Chiu C.-C., Chen H.-R. (2013). Calreticulin, an endoplasmic reticulum-resident protein, is highly expressed and essential for cell proliferation and migration in oral squamous cell carcinoma. Oral Oncol..

[17] Yi L., Shan J., Chen X., Li G., Li L., Tan H. (2016). Involvement of calreticulin in cell proliferation, invasion and differentiation in diallyl disulfide-treated HL-60 cells. Oncol. Lett..

[18] Gold L.I., Rahman M., Blechman K.M., Greives M.R., Churgin S., Michaels J. (2006). Overview of the role for calreticulin in the enhancement of wound healing through multiple biological effects. J. Investig. Dermatol. Symp. Proc..

[19] Papp S., Fadel M.P., Kim H., McCulloch C.A., Opas M. (2007). Calreticulin affects fibronectin-based cell-substratum adhesion via the regulation of c-Src activity. J. Biol. Chem..

[20] Ogden C.A., deCathelineau A., Hoffmann P.R., Bratton D., Ghebrehiwet B., Fadok V.A. (2001). C1q and mannose-binding lectin engagement of cell surface calreticulin and CD91 initiates macropinocytosis and uptake of apoptotic cells. J. Exp. Med..

[21] Martins I., Kepp O., Galluzzi L., Senovilla L., Schlemmer F., Adjemian S. (2010). Surface-exposed calreticulin in the interaction between dying cells and phagocytes. Ann. N. Y. Acad. Sci..

[22] Gardai S.J., McPhillips K.A., Frasch S.C., Janssen W.J., Starefeldt A., Murphy-Ullrich J.E. (2005). Cell-surface calreticulin initiates clearance of viable or apoptotic cells through trans-activation of LRP on the phagocyte. Cell.

[23] Chao M.P., Jaiswal S., Weissman-Tsukamoto R., Alizadeh A.A., Gentles A.J., Volkmer J. (2010). Calreticulin is the dominant pro-phagocytic signal on multiple human cancers and is counterbalanced by CD47. Sci. Transl. Med..

[24] Obeid M., Tesniere A., Ghiringhelli F., Fimia G.M., Apetoh L., Perfettini J.-L. (2007). Calreticulin exposure dictates the immunogenicity of cancer cell death. Nat. Med..

[25] Zamanian M., Veerakumarasivam A., Abdullah S., Rosli R. (2013). Calreticulin and cancer. Pathol. Oncol. Res..

[26] Alur M., Nguyen M.M., Eggener S.E., Jiang F., Dadras S.S., Stern J. (2009). Suppressive roles of calreticulin in prostate cancer growth and metastasis. Am. J. Pathol..

[27] Zamanian M., Qader Hamadneh L.A., Veerakumarasivam A., Abdul Rahman S., Shohaimi S., Rosli R. (2016). Calreticulin mediates an invasive breast cancer phenotype through the transcriptional dysregulation of p53 and MAPK pathways. Cancer Cell Int..

[28] Shi F., Shang L., Pan B.-Q., Wang X.-M., Jiang Y.-Y., Hao J.-J. (2014). Calreticulin promotes migration and invasion of esophageal cancer cells by upregulating neuropilin-1 expression via STAT5A. Clin. Cancer Res..

[29] Borg R.J., Samson A.L., Au A.E.-L., Scholzen A., Fuchsberger M., Kong Y.Y. (2015). Dendritic cell-mediated phagocytosis but not immune activation is enhanced by plasmin. PLoS One.

[30] Sheng W., Chen C., Dong M., Zhou J., Liu Q., Dong Q. (2014). Overexpression of calreticulin contributes to the development and progression of pancreatic cancer. J. Cell. Physiol..

[31] Andreasen P.A., Egelund R., Petersen H.H. (2000). The plasminogen activation system in tumor growth, invasion, and metastasis. Cell. Mol. Life Sci..

[32] Kumari S., Malla R. (2015). New insight on the role of plasminogen receptor in cancer progression. Cancer Growth Metastasis.

[33] Didiasova M., Wujak L., Wygrecka M., Zakrzewicz D. (2014). From plasminogen to plasmin: role of plasminogen receptors in human cancer. Int. J. Mol. Sci..

[34] Wu M., Massaeli H., Durston M., Mesaeli N. (2007). Differential expression and activity of matrix metalloproteinase-2 and -9 in the calreticulin deficient cells. Matrix Biol..

[35] Baramova E.N., Bajou K., Remacle A., L’Hoir C., Krell H.W., Weidle U.H. (1997). Involvement of PA/plasmin system in the processing of pro-MMP-9 and in the second step of pro-MMP-2 activation. FEBS Lett..

[36] Legrand C., Polette M., Tournier J.M., de Bentzmann S., Huet E., Monteau M. (2001). uPA/plasmin system-mediated MMP-9 activation is implicated in bronchial epithelial cell migration. Exp. Cell Res..

[37] Miles L.A., Plow E.F., Waisman D.M., Parmer R.J. (2012). Plasminogen receptors. J. Biomed. Biotechnol..

[38] Plow E.F., Doeuvre L., Das R. (2012). So many plasminogen receptors: why?. J. Biomed. Biotechnol..

[39] Miles L.A., Parmer R.J. (2013). Plasminogen receptors: the first quarter century. Semin. Thromb. Hemost..

[40] Godier A., Hunt B.J. (2013). Plasminogen receptors and their role in the pathogenesis of inflammatory, autoimmune and malignant disease. J. Thromb. Haemost..

[41] Bydoun M., Waisman D.M. (2014). On the contribution of S100A10 and annexin A2 to plasminogen activation and oncogenesis: an enduring ambiguity. Future Oncol..

[42] Madureira P.A., O’Connell P.A., Surette A.P., Miller V.A., Waisman D.M. (2012). The biochemistry and regulation of S100A10: a multifunctional plasminogen receptor involved in oncogenesis. J. Biomed. Biotechnol..

[43] Galántai R., Módos K., Fidy J., Kolev K., Machovich R. (2006). Structural basis of the cofactor function of denatured albumin in plasminogen activation by tissue-type plasminogen activator. Biochem. Biophys. Res. Commun..

[44] Khanna N.C., Tokuda M., Waisman D.M. (1986). Conformational changes induced by binding of divalent cations to calregulin. J. Biol. Chem..

[45] Wijeyesakere S.J., Gafni A.A., Raghavan M. (2011). Calreticulin is a thermostable protein with distinct structural responses to different divalent cation environments. J. Biol. Chem..

[46] Siddiq M.M., Tsirka S.E. (2004). Modulation of zinc toxicity by tissue plasminogen activator. Mol. Cell. Neurosci..

[47] Boxrud P.D., Verhamme I.M., Fay W.P., Bock P.E. (2001). Streptokinase triggers conformational activation of plasminogen through specific interactions of the amino-terminal sequence and stabilizes the active zymogen conformation. J. Biol. Chem..

[48] Medcalf R.L. (2017). Fibrinolysis: from blood to the brain. J. Thromb. Haemost..

[49] Wiman B., Andersson T., Hallqvist J., Reuterwall C., Ahlbom A., deFaire U. (2000). Plasma levels of tissue plasminogen activator/plasminogen activator inhibitor-1 complex and von Willebrand factor are significant risk markers for recurrent myocardial infarction in the Stockholm Heart Epidemiology Program (SHEEP) study. Arterioscler. Thromb. Vasc. Biol..

[50] Nangalia J., Massie C.E., Baxter E.J., Nice F.L., Gundem G., Wedge D.C. (2013). Somatic CALR mutations in myeloproliferative neoplasms with nonmutated JAK2. N. Engl. J. Med..

[51] Klampfl T., Gisslinger H., Harutyunyan A.S., Nivarthi H., Rumi E., Milosevic J.D. (2013). Somatic mutations of calreticulin in myeloproliferative neoplasms. N. Engl. J. Med..

[52] Čiplys E., Paškevičius T., Žitkus E., Bielskis J., Ražanskas R., Šneideris T. (2021). Mapping human calreticulin regions important for structural stability. Biochim. Biophys. Acta Proteins Proteom..

[53] Mesaeli N., Nakamura K., Zvaritch E., Dickie P., Dziak E., Krause K.H. (1999). Calreticulin is essential for cardiac development. J. Cell Biol..

[54] Nakamura K., Zuppini A., Arnaudeau S., Lynch J., Ahsan I., Krause R. (2001). Functional specialization of calreticulin domains. J. Cell Biol..

[55] Goicoechea S., Pallero M.A., Eggleton P., Michalak M., Murphy-Ullrich J.E. (2002). The anti-adhesive activity of thrombospondin is mediated by the N-terminal domain of cell surface calreticulin. J. Biol. Chem..

[56] Gao B., Adhikari R., Howarth M., Nakamura K., Gold M.C., Hill A.B. (2002). Assembly and antigen-presenting function of MHC class I molecules in cells lacking the ER chaperone calreticulin. Immunity.

[57] Azuma Y., Suzuki K., Higai K., Matsumoto K., Tada S. (2020). Biphasic increases of cell surface calreticulin following treatment with mitoxantrone. Biol. Pharm. Bull..

[58] Ostwald T.J., MacLennan D.H. (1974). Isolation of a high affinity calcium-binding protein from sarcoplasmic reticulum. J. Biol. Chem..

[59] Fliegel L., Burns K., MacLennan D.H., Reithmeier R.A., Michalak M. (1989). Molecular cloning of the high affinity calcium-binding protein (calreticulin) of skeletal muscle sarcoplasmic reticulum. J. Biol. Chem..

[60] Macer D.R., Koch G.L. (1988). Identification of a set of calcium-binding proteins in reticuloplasm, the luminal content of the endoplasmic reticulum. J. Cell Sci..

[61] Treves S., Vukcevic M., Maj M., Thurnheer R., Mosca B., Zorzato F. (2009). Minor sarcoplasmic reticulum membrane components that modulate excitation–contraction coupling in striated muscles. J. Physiol..

[62] Barone V., Randazzo D., Del Re V., Sorrentino V., Rossi D. (2015). Organization of junctional sarcoplasmic reticulum proteins in skeletal muscle fibers. J. Muscle Res. Cell Motil..

[63] Leberer E., Charuk J.H., Green N.M., MacLennan D.H. (1989). Molecular cloning and expression of cDNA encoding a lumenal calcium-binding glycoprotein from sarcoplasmic reticulum. Proc. Natl. Acad. Sci. U. S. A..

[64] Hofmann S.L., Brown M.S., Lee E., Pathak R.K., Anderson R.G., Goldstein J.L. (1989). Purification of a sarcoplasmic reticulum protein that binds Ca2+ and plasma lipoproteins. J. Biol. Chem..

[65] Damiani E., Margreth A. (1991). Subcellular fractionation to junctional sarcoplasmic reticulum and biochemical characterization of 170 kDa Ca(2+)- and low-density-lipoprotein-binding protein in rabbit skeletal muscle. Biochem. J..

[66] Campbell K.P., MacLennan D.H., Jorgensen A.O. (1983). Staining of the Ca2+-binding proteins, calsequestrin, calmodulin, troponin C, and S-100, with the cationic carbocyanine dye “Stains-all”. J. Biol. Chem..

[67] Leberer E., Timms B.G., Campbell K.P., MacLennan D.H. (1990). Purification, calcium-binding properties, and ultrastructural localization of the 53,000- and 160,000 (sarcalumenin)-dalton glycoproteins of the sarcoplasmic reticulum. J. Biol. Chem..

[68] Bharadwaj A.G., Miller V.A., Waisman D.M. (2019). The high-affinity calcium-binding protein is not calreticulin. J. Mol. Biol. Ther..

[69] MacLennan D.H., Ostwald T.J., Stewart P.S. (1974). Structural components of the sarcoplasmic reticulum membrane. Ann. N. Y. Acad. Sci..

[70] Khanna N.C., Tokuda M., Waisman D.M. (1987). Comparison of calregulins from vertebrate livers. Biochem. J..

[71] Smith M.J., Koch G.L. (1989). Multiple zones in the sequence of calreticulin (CRP55, calregulin, HACBP), a major calcium binding ER/SR protein. EMBO J..

[72] Lewis M.J., Mazzarella R.A., Green M. (1985). Structure and assembly of the endoplasmic reticulum. The synthesis of three major endoplasmic reticulum proteins during lipopolysaccharide-induced differentiation of murine lymphocytes. J. Biol. Chem..

[73] Van P.N., Peter F., Söling H.D. (1989). Four intracisternal calcium-binding glycoproteins from rat liver microsomes with high affinity for calcium. No indication for calsequestrin-like proteins in inositol 1,4,5-trisphosphate-sensitive calcium sequestering rat liver vesicles. J. Biol. Chem..

[74] Feng M., Marjon K.D., Zhu F., Weissman-Tsukamoto R., Levett A., Sullivan K. (2018). Programmed cell removal by calreticulin in tissue homeostasis and cancer. Nat. Commun..

[75] Ellgaard L., Frickel E.-M. (2003). Calnexin, calreticulin, and ERp57: teammates in glycoprotein folding. Cell Biochem. Biophys..

[76] Lum R., Ahmad S., Hong S.J., Chapman D.C., Kozlov G., Williams D.B. (2016). Contributions of the lectin and polypeptide binding sites of calreticulin to its chaperone functions in vitro and in cells. J. Biol. Chem..

[77] Kozlov G., Pocanschi C.L., Rosenauer A., Bastos-Aristizabal S., Gorelik A., Williams D.B. (2010). Structural basis of carbohydrate recognition by calreticulin. J. Biol. Chem..

[78] Longstaff C., Kolev K. (2015). Basic mechanisms and regulation of fibrinolysis. J. Thromb. Haemost..

[79] Das R., Ganapathy S., Settle M., Plow E.F. (2014). Plasminogen promotes macrophage phagocytosis in mice. Blood.

[80] Rosenwald M., Koppe U., Keppeler H., Sauer G., Hennel R., Ernst A. (2012). Serum-derived plasminogen is activated by apoptotic cells and promotes their phagocytic clearance. J. Immunol..

[81] Baker S.K., Strickland S. (2020). A critical role for plasminogen in inflammation. J. Exp. Med..

[82] Wyganowska-Świątkowska M., Tarnowski M., Murtagh D., Skrzypczak-Jankun E., Jankun J. (2019). Proteolysis is the most fundamental property of malignancy and its inhibition may be used therapeutically (review). Int. J. Mol. Med..

[83] McMahon B.J., Kwaan H.C. (2015). Components of the plasminogen-plasmin system as biologic markers for cancer. Adv. Exp. Med. Biol..

[84] Miles L.A., Hawley S.B., Baik N., Andronicos N.M., Castellino F.J., Parmer R.J. (2005). Plasminogen receptors: the sine qua non of cell surface plasminogen activation. Front. Biosci..

[85] Felez J., Miles L.A., Fabregas P., Jardi M., Plow E.F., Lijnen R.H. (1996). Characterization of cellular binding sites and interactive regions within reactants required for enhancement of plasminogen activation by tPA on the surface of leukocytic cells. Thromb. Haemost..

[86] Romagnuolo R., Marcovina S.M., Boffa M.B., Koschinsky M.L. (2014). Inhibition of plasminogen activation by apo(a): role of carboxyl-terminal lysines and identification of inhibitory domains in apo(a). J. Lipid Res..

[87] Horne M.K., Merryman P.K., Cullinane A.M. (2005). Plasminogen interaction with platelets: the importance of carboxyterminal lysines. Thromb. Res..

[88] Plow E.F., Herren T., Redlitz A., Miles L.A., Hoover-Plow J.L. (1995). The cell biology of the plasminogen system [review]. FASEB J..

[89] Ranson M., Andronicos N.M., O’Mullane M.J., Baker M.S. (1998). Increased plasminogen binding is associated with metastatic breast cancer cells: differential expression of plasminogen binding proteins. Br. J. Cancer.

[90] Wijeyesakere S.J., Rizvi S.M., Raghavan M. (2013). Glycan-dependent and -independent interactions contribute to cellular substrate recruitment by calreticulin. J. Biol. Chem..

[91] Ling S., Pi X., Holoshitz J. (2007). The rheumatoid arthritis shared epitope triggers innate immune signaling via cell surface calreticulin. J. Immunol..

[92] Moreau C., Bally I., Chouquet A., Bottazzi B., Ghebrehiwet B., Gaboriaud C. (2016). Structural and functional characterization of a single-chain form of the recognition domain of complement protein C1q. Front. Immunol..

[93] McDonnell J.M., Jones G.E., White T.K., Tanzer M.L. (1996). Calreticulin binding affinity for glycosylated laminin. J. Biol. Chem..

[94] Sakono M., Seko A., Takeda Y., Ito Y. (2014). PDI family protein ERp29 forms 1:1 complex with lectin chaperone calreticulin. Biochem. Biophys. Res. Commun..

[95] Kassam G., Le B.H., Choi K.S., Kang H.M., Fitzpatrick S.L., Louie P. (1998). The p11 subunit of the annexin II tetramer plays a key role in the stimulation of t-PA-dependent plasminogen activation. Biochemistry.

[96] Johnsen L.B., Ravn P., Berglund L., Petersen T.E., Rasmussen L.K., Heegaard C.W. (1998). A refined kinetic analysis of plasminogen activation by recombinant bovine tissue-type plasminogen activator indicates two interconvertible activator forms. Biochemistry.

[97] Basu S., Binder R.J., Ramalingam T., Srivastava P.K. (2001). CD91 is a common receptor for heat shock proteins gp96, hsp90, hsp70, and calreticulin. Immunity.

[98] Pausz C., Mawas R., Unseld M., Chilla A., Novotny R., Prager G.W. (2014). uPAR directly interacts with LDLR-like proteins to induce angiogenic endothelial cell behavior. Blood.

[99] Law R.H.P., Caradoc-Davies T., Cowieson N., Horvath A.J., Quek A.J., Encarnacao J.A. (2012). The X-ray crystal structure of full-length human plasminogen. Cell Rep..

[100] Miles L.A., Ny L., Wilczynska M., Shen Y., Ny T., Parmer R.J. (2021). Plasminogen receptors and fibrinolysis. Int. J. Mol. Sci..

[101] Gong Y., Kim S.O., Felez J., Grella D.K., Castellino F.J., Miles L.A. (2001). Conversion of Glu-plasminogen to Lys-plasminogen is necessary for optimal stimulation of plasminogen activation on the endothelial cell surface. J. Biol. Chem..

[102] Choi K.S., Fogg D.K., Yoon C.S., Waisman D.M. (2003). P11 regulates extracellular plasmin production and invasiveness of HT1080 fibrosarcoma cells. FASEB J..

[103] Kassam G., Choi K.S., Ghuman J., Kang H.M., Fitzpatrick S.L., Zackson T. (1998). The role of annexin II tetramer in the activation of plasminogen. J. Biol. Chem..

[104] Nieuwenhuizen W., Voskuilen M., Vermond A., HOEGEE-de NOBEL B., Traas D.W. (1988). The influence of fibrin(ogen) fragments on the kinetic parameters of the tissue-type plasminogen-activator-mediated activation of different forms of plasminogen. Eur. J. Biochem..

[105] Panaretakis T., Joza N., Modjtahedi N., Tesniere A., Vitale I., Durchschlag M. (2008). The co-translocation of ERp57 and calreticulin determines the immunogenicity of cell death. Cell Death Differ..

[106] Ranby M. (1982). Studies on the kinetics of plasminogen activation by tissue plasminogen activator. Biochim. Biophys. Acta.

