# Supplementary material for: Identification and characterization of calreticulin as a novel plasminogen receptor
Source: J Biol Chem. 2023 Nov 17;300(1):105465. doi: 10.1016/j.jbc.2023.105465 (PMC10770727; doi:10.1016/j.jbc.2023.105465)
Supplement: Supporting Figures S1–S5 [file mmc1.docx]

Article: Identification and Characterization of Calreticulin as a Novel Plasminogen Receptor.

Alamelu G. Bharadwaj^1,2^, Gillian C. Okura^1^, John W. Woods^2^, Erica A. Allen^1^, Victoria A. Miller^1^, Emma Kempster^1^, Mark A. Hancock^3^, Shashi Gujar^1^, Rimantas Slibinskas^4^, and David M. Waisman^1,2^*

Affiliated institutions:

^1^Departments of Pathology, ^2^Biochemistry and Molecular Biology, Dalhousie University, Halifax, Nova Scotia, B3H 1X5, Canada

^3^McGill SPR-MS Facility, McGill University, Montréal, Québec, Canada.

^4^ Institute of Biotechnology, Life Sciences Center, Vilnius University, Saulėtekio 7, LT-10257 Vilnius, Lithuania. Electronic address: rimantas.slibinskas@bti.vu.lt.

*To whom correspondence should be addressed: David M. Waisman. Department of Pathology and the Department of Biochemistry and Molecular Biology, Faculty of Medicine, Sir Charles Tupper Medical Building, Dalhousie University, Halifax, Nova Scotia, Canada B3H 1X5, Tel.: (902) 494-1803; Fax: (902) 494-1355; E-mail: david.waisman@dal.ca

**Supporting Information**
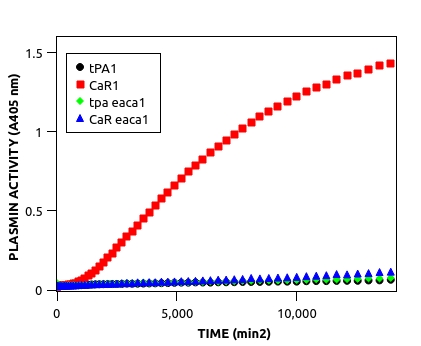


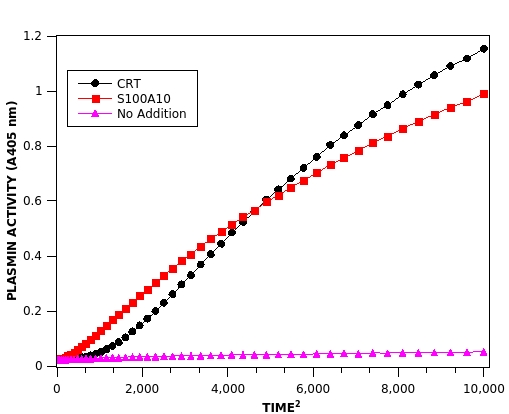


**Figure S1. Stimulation of plasmin generation by human recombinant CRT. (A) Time course of stimulation of tPA-dependent plasmin generation by recombinant human CRT.** t-PA (0.1 nM) was incubated at 37 °C in buffer A (50 mM Tris-HCl (pH 7.4), 50 mM NaCl, and 5 mM CaCl2) with 360 μM plasmin substrate in the absence (circles) or presence (squares) of 0.5 µM recombinant human CRT or 0.5 µM purified bovine lung S100A10 (p11) (triangles). The reaction was initiated by the addition of 0.16 μM Glu-plasminogen, and the reaction was monitored at 405 nm. The data is plotted as A405 nm vs. t^2^. **(B) Inhibition of CRT activity by ε-ACA.** tPA was incubated in the presence (filled squares) or absence (filled circles) of 0.5 µM human recombinant CRT as described above. In some experiments, 10 mM ε-ACA was added before the reaction was initiated (open symbols). The rates are plotted as A405 nm vs. t^2^


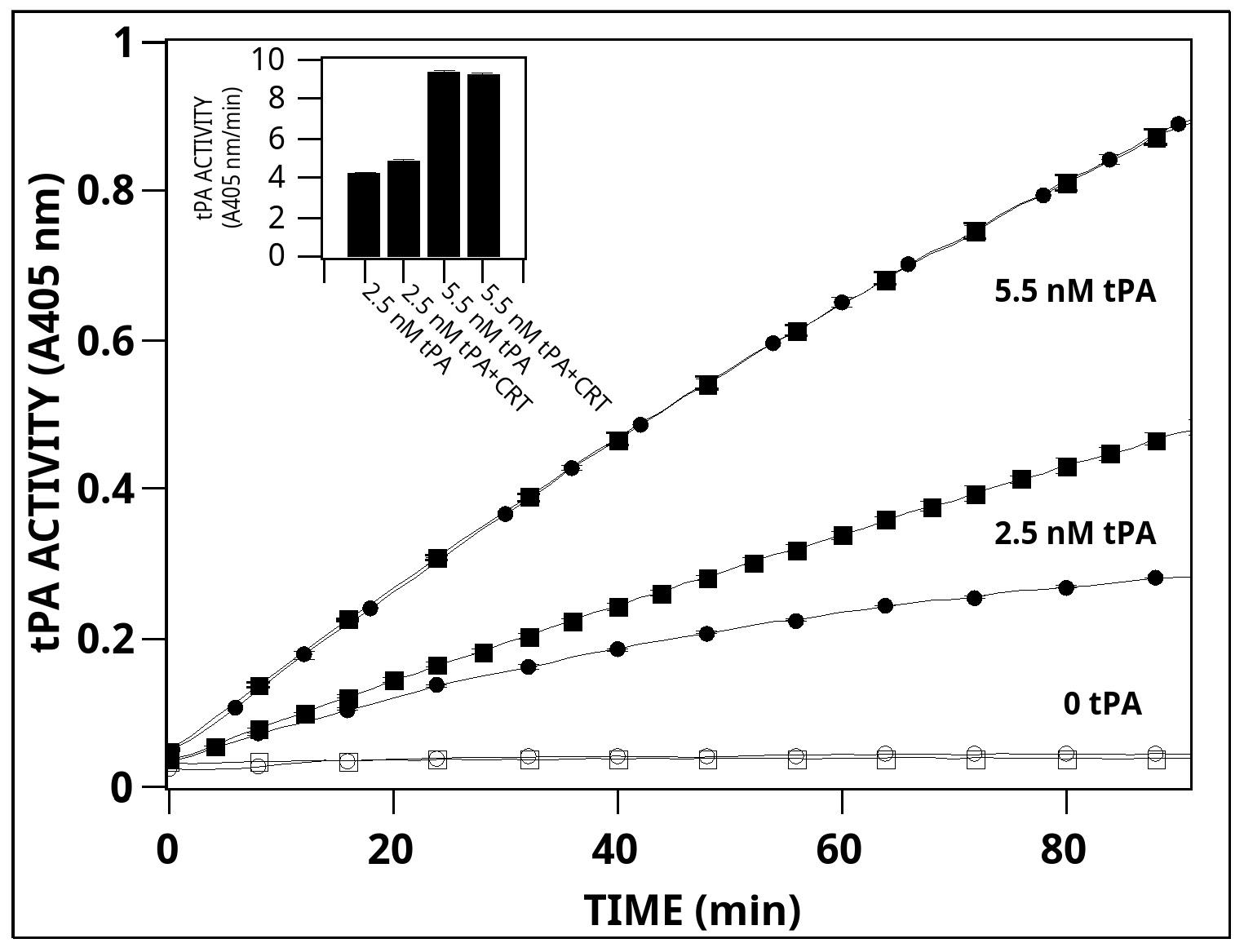


Figure S2. **t-PA amidolytic activity was minimally stimulated by CRT.** The tPA amidolytic activity was directly measured at 37 °C in 0.2 ml of a reaction mixture consisting of 100 mM Tris-HCl (pH 8.4), 106 mM NaCl, and 0.1 g/L Triton X-100 and 8 nM of the tPA substrate, S-2288 H-D-Isoleucyl-L-prolyl-L-arginine-p-nitroaniline dihydrochloride (Diapharma Group, OH, USA). The reaction was monitored at 405 nm, and the reaction rate was calculated using linear regression analysis of plots of A405 nm versus time (in minutes). Typically, results are representative of at least three separate experiments performed in triplicate.


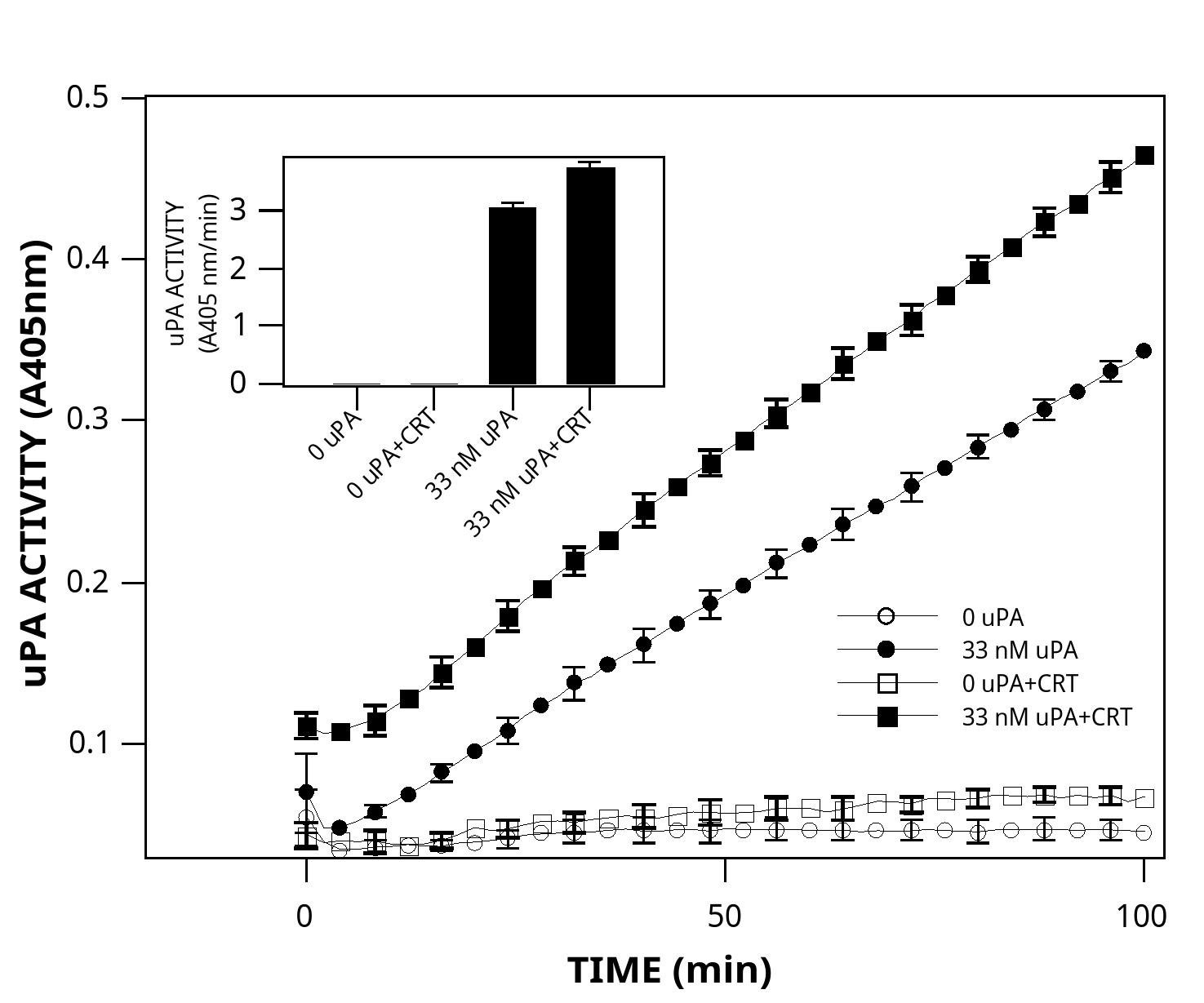


Figure S3 **The uPA amidolytic activity was not stimulated by CRT.** uPA amidolytic activity was measured in 0.2 ml of a reaction mixture consisting of 50 mM Tris-HCl (pH 7.4), 50 mM NaCl, 5 mM CaCl2, and 500 μM uPA substrate, Diapharma 444-25). The reaction was monitored at 405 nm, and the reaction rate was calculated using linear regression analysis of plots of A405 nm versus time (in minutes). Typically, results are representative of at least three separate experiments performed in triplicate.


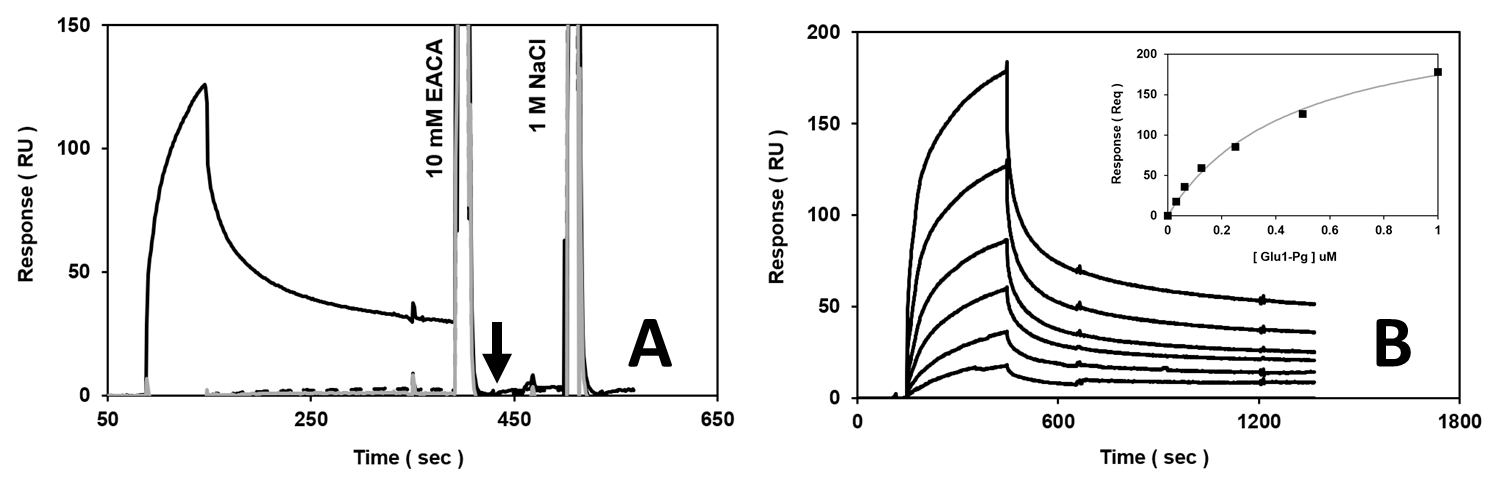


Figure S4. **Specific, dose-dependent binding between S100A10 and Glu-plasminogen is lysine-dependent, as assessed by SPR.** (A) representative specificity of buffer blank (grey line, baseline), 500 nM BSA (dashed black line, negative control), or 500 nM Glu-plasminogen (solid black line) injected over immobilized S100A10 surfaces at 50 μ/min (1 min association + 3 min dissociation); to remove residually-bound plasminogen back to baseline (black arrow), immobilized S100A10 surfaces were readily regenerated using a lysine analog (spike #1, 10 mM EACA) before the added high salt pulse (spike #2, 1M NaCl). B, representative kinetics for increasing Glu-plasminogen concentrations (0, 0.031, 0.062, 0.125, 0.25, 0.5, and 1 uM) titrated over immobilized S100A10 at 50 uL/min (5 min association + 15 min dissociation); inset, dose-dependent isotherm for Glu-plasminogen binding to S100A10 (black symbols) was subjected to non-linear regression analysis (grey line) to predict the apparent equilibrium dissociation constant (KD = 0.5 +/- 0.07 mM).


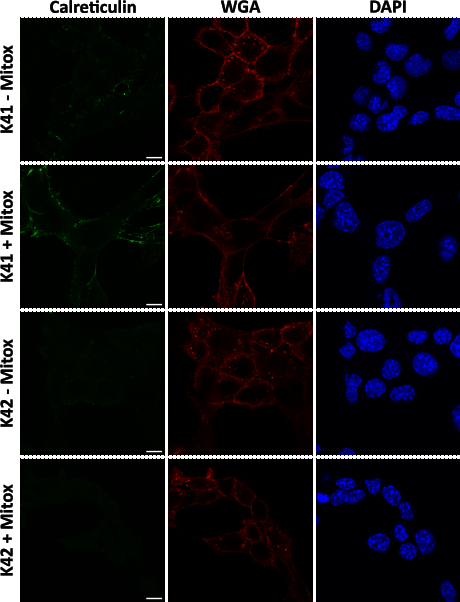


Figure S5. **Mitoxantrone treatment increases cell surface localization of CRT in the CRT-WT expressing mouse embryonic fibroblasts (MEFs).** Mouse embryonic fibroblasts (MEFs) K41 (CRT-wild-type) and K42 (CRT-Knock-out) were seeded on poly-L-lysine treated coverslips (Carl Zeiss, Canada) overnight. The cells were then treated with 5 µM Mitoxantrone (Mitox) for 4 hours. Cell surface CRT was stained using anti-CRT antibody (Ab2907, Abcam). Briefly, the cells were washed three times with Phosphate Buffered Saline (PBS, Thermo Fisher Scientific, Canada) and stained with anti-CRT primary antibody (1:100) in PBS with 2% Fetal Bovine. Serum (FBS) for 30 mins at 4^0^C. This is followed by washing and fixing in 4% Paraformaldehyde (PFA) for 15 minutes at RT. The cells were then incubated with anti-rabbit Alexa-488 secondary (Thermo Fisher Scientific, Canada, 1:500) (green) and wheat germ agglutinin (Thermo Fisher Scientific, Canada, 5 µg/ml) (Red) for 30 mins at 4^0^C in the dark. The cells were washed following incubation and incubated with DAPI (blue) for staining the nucleus, and finally mounted with Prolong Gold (Thermo Fisher Scientific, Canada), cured and imaged using Leica TCS SP8 confocal (Dalhousie University). Scale bar – 10 µM.
